# Simple and Efficient Synthesis of Diamino Derivatives of bis-1,2,4-oxadiazole *via* Tandem Staudinger/aza-Wittig Reaction

**DOI:** 10.2174/1570179420666221006113032

**Published:** 2023-04-19

**Authors:** Hai Xie, Qing-Qing Hu, Ya-Li Zhang, Xiu-Ting Qin, Lu Li

**Affiliations:** 1 College of Chemistry and Chemical Engineering, Shanxi Datong University, Datong, People’s Republic of China

**Keywords:** Diamino derivatives of bis-1,2,4-oxadiazole, Staudinger reaction, aza-Wittig reaction, diaziglyoxime, diazioxalimides, heterocyclic compounds

## Abstract

Two efficient, scalable routes to bis-1,2,4-oxadiazole have been developed by tandem Staudinger/aza-Wittig reaction from the same starting material diaziglyoxime, isocyanates and triphenylphosphonium in good yields.

***Background*:** Two convenient and efficient routes for synthesizing diamino derivatives of bis-1,2,4-oxadiazoles were described.

***Objective*:** This study provides a simple protocol for the synthesis of bis-1,2,4-oxadiazoles.

***Methods*:** The two procedures were based on a tandem Staudinger/aza-Wittig reaction from the same starting material of diaziglyoxime, isocyanates and triphenylphosphonium.

***Results*:** In synthesis method I, diaziglyoxime **1** was treated with various aromatic or aliphatic isocyanates to give diazioxalimides **2** a high yield. Diazioxalimides **2** reacted with Ph_3_P to produce the iminophosphoranes **4**; the reaction was directly heated from room temperature to 115°C to get the desired diamino derivatives of bis-1,2,4-oxadiazole **4** in 72-92% yields. In synthesis method II, the same target compounds **4** were synthesized in a one-pot reaction by Ph_3_P and aromatic or aliphatic isocyanates in toluene for 10 h under 115 °C in 53-71% yields.

***Conclusion*:** The two procedures provide proficient methods of making nitrogen-containing heterocyclic rings. The structures of target compounds 4 were identified by IR, ^1^HNMR, ^13^CNMR and HRMS.

## 1 INTRODUCTION

**Fig. (1):**
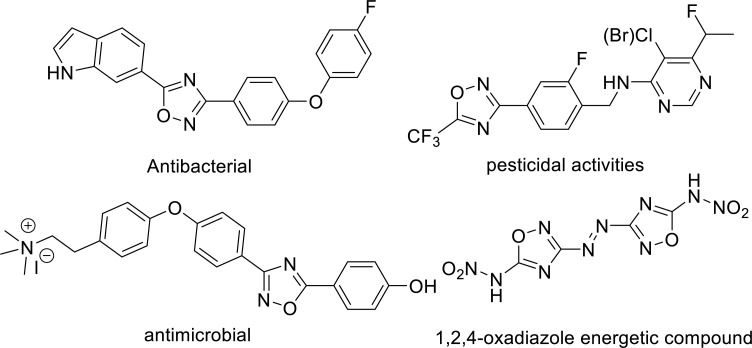
Representative application of 1,2,4-oxadiazoles.

Oxadiazoles are one of the most important heterocyclic compounds with desirable biological or functional material properties, which are used widely in medicine, pesticidal chemistry and other fields as a stable bioisosterism of esters or amides. As a key member of the oxadiazole family, drugs containing a 1,2,4-oxadiazole core are associated with a diverse range of pharmacological and biological activities, such as anticancer [1, 2], antimicrobial [3], antibacterial activity [4], anti-inflammatory activity [5], pesticidal activities [6] and HDAC inhibitors for Huntington’s disease [7]. In addition, 5-amino-1,2,4-oxadiazoles are used as a very promising energetic component for the preparation of high-energy-density materials (HEDMs) [8, 9] (Fig. **1**).

Although there were many synthetic methods of oxadiazoles [10, 11], there were only a few pieces of literature on synthesizing bisoxadiazole rings [12-14]. The aza-Wittig reaction becomes a simple, efficient, inexpensive tool for constructing nitrogen-containing heterocyclic rings [15, 16].

A large number of five-, six- and seven-membered heterocycles containing a nitrogen heteroatom could be synthesized *via* the aza-Wittig reaction in reasonable yields.

Recently, we have synthesized a series of oxazoles [17-19], benzodiazepines [20] and imidazoles [21] using various azides as starting material *via* the aza-Wittig reaction. Herein, we described two synthetic routes of bis-1,2,4-oxadiazoles *via* tandem Staudinger/aza-Wittig reaction of diaziglyoxime and diazioxalimides promoted by PPh_3_.

## 2 MATERIAL AND METHODS

Dichloroglyoxime, sodium azide, triphenylphosphine, isocyanates, anhydrous sodium sulfate and the common solvents are commercially available analytical purity and can be used directly without purification. Toluene (C_6_H_5_CH_3_) and chloroform (CHCl_3_) were dried with anhydrous calcium chloride for one week.

All melting points were determined on an X-4 model melting point apparatus and uncorrected previously. IR spectra were recorded on a Perkin Elmer-Spectrum One spectrometer as KBr pellets and reported in cm^-1^. ^1^H NMR and ^13^C NMR spectra were acquired on AVANCE NEO 500M spectrometer (500 and 125 MHz, respectively) in DMSO and resonances relative to TMS. High-resolution mass spectra (HRMS) were performed by an Agilent 6224 TOF LC/MS spectrometer. Monitoring of the reaction progress and assessment of the purity of synthesized compounds was done by TLC on Silica gel (HSGF254) plates, and Silica gel (200-300 mesh) was used for short column chromatography.

### 2.1 Synthesis of 2a-2h

Diaziglyoxime **1** (1.02 g, 6 mmol) and various aromatic or aliphatic isocyanates (12 mmol) were dissolved in chloroform (15 mL), and the mixture was stirred for about 4 h at room temperature (TLC showed complete conversion). The reaction mixture was poured into 50 mL water and stirred for a few minutes, and a large amount of yellow solid was precipitated from the water. The formed precipitate was filtered off, washed with a small amount of ethanol, and crystallized from *n*-hexane/ether (6:1) to give the diazioxalimides **2a-2j** as yellow solids.

#### 2.1.1 N^'1^,N^'2^-bis((phenylcarbamoyl)oxy)oxalimidoyl diazide (2a)

Yellow solid (0.82 g, 82%) m.p.240°C; IR (KBr):3384, 2128, 1762, 1441, 1294, 1243, 1172 cm^-1^; ^1^H NMR (500 MHz, DMSO) δ 8.66 (s, NH, 2H), δ 7.45 (d, J = 7.3 Hz, ArH, 4H), 7.29-7.26 (t, ArH, 4H), 6.98-6.95 (t, ArH, 2H); ^13^C NMR (125 MHz, DMSO) δ 150.28, 142.03, 138.11, 129.16, 128.99, 123.68, 119.07.

#### 2.1.2 N^'1^,N^'2^-bis((p-tolylcarbamoyl)oxy)oxalimidoyl diazide (2b)

Yellow solid (0.77 g, 77%) m.p.:275°C; IR (KBr): 3412, 2160, 1767, 1406, 1303,1179, 1077 cm^-1^; ^1^H NMR (500 MHz, DMSO) δ 8.49 (s, NH, 2H), 7.32 (d, J = 8.5, ArH, 4H), 7.07 (d, J = 8.2 Hz, ArH, 4H), 2.23 (s, CH_3_, 6H); ^13^C NMR (125 MHz, DMSO) δ 152.78, 137.39, 130.65, 129.31, 118.38, 20.48.

#### 2.1.3 N^'1^,N^'2^-bis(((4-chlorophenyl)carbamoyl)oxy)oxalimi-doyl diazi (2c)

Yellow solid (0.71, 71%) m.p.:285-290°C; IR (KBr): 3294, 2107, 1893, 1631, 1590, 1490, 1280, 1084 cm^-1^; ^1^H NMR (500 MHz, DMSO) δ 8.85 (s, NH, 2H), 7.61-7.39 (m, ArH, 4H), 7.39-7.01(m, ArH, 4H). ^13^C NMR (125 MHz, DMSO) δ 152.36, 138.56, 128.63, 125.53, 119.85.

#### 2.1.4 N^'1^,N^'2^-bis(((4-fluorophenyl)carbamoyl)oxy)oxalimid-oyl diazide (2d)

Yellow solid (0.74 g, 74%) m.p.:234-245°C; IR (KBr): 3401, 2124, 1774, 1611, 1597, 1409, 1253, 1171 cm^-1^; ^1^H NMR (500 MHz, DMSO) δ 8.68 (s, NH, 2H), 7.44 (d, ArH, 4H), 7.10 (d, J = 8.9 Hz, ArH, 4H). ^13^C NMR (125 MHz, DMSO) δ 158.46, 156.56, 152.89, 136.17, 136.15, 120.21, 120.15, 115.50, 115.32.

#### 2.1.5 N^'1^,N^'2^-bis(((3-chlorophenyl)carbamoyl)oxy)oxalimi-doyl diazide (2e)

Yellow solid (0.62 g, 62%) m.p.:251°C; IR (KBr): 3368, 3306, 2155, 1723, 1617,1522, 1497, 1278, 1215, 1006 cm^-1^; ^1^H NMR (500 MHz, DMSO) δ 8.97 (s, NH, 2H), 7.71 (s, ArH, 2H), 7.48-7.14 (m, ArH, 4H), 7.04 (d, J = 7.7Hz, ArH, 2H). ^13^C NMR (125 MHz, DMSO) δ 152.41, 141.15, 133.37, 130.54, 121.86, 117.93, 116.99.

#### 2.1.6 N^'1^,N^'2^-bis(((4-bromophenyl)carbamoyl)oxy)oxalim-idoyl diazide (2f)

Yellow solid (0.62 g, 79%) m.p. 194-196°C; IR (KBr): 3387,3300,2150,1767,1634,1595, 1489, 1239, 1073 cm^-1^; ^1^H NMR (500 MHz, DMSO) δ10.49 (s, NH 2H), 7.54-7.48 (m, ArH, 8H). ^13^C NMR (125 MHz, DMSO) δ150.52, 142.58, 137.90, 132.31, 121.28, 120.74, 115.73.

#### 2.1.7 N^'1^,N^'2^-bis(((4-nitrophenyl)carbamoyl)oxy)oxalim-idoyl diazide (2g)

Yellow solid(0.68 g, 81%)m.p. >300°C;IR (KBr): 3366, 2128, 1733, 1616, 1554, 1497, 1248, 1177 cm^-1^; ^1^H NMR (500 MHz, DMSO) δ9.67 (s, NH, 2H), 8.23 (d, ArH, 4H), 7.73 (d, ArH, 4H). ^13^C NMR (125 MHz, DMSO)δ151.82, 145.88, 141.70, 125.35, 118.16.

#### 2.1.8 N^'1^,N^'2^-bis((cyclohexylcarbamoyl)oxy)oxalimidoyl diazide (2h)

Yellow solid (0.56 g, 63%) m.p.230°C; IR (KBr): 3368, 2155, 1723, 1617, 1522, 1497, 1278, 1215 cm^-1^; ^1^H NMR (500 MHz, DMSO) δ 5.56 (s, NH, 2H), 1.72-1.48 (m, 11H), 1.28-0.99 (m, 11H). ^13^C NMR (125 MHz, DMSO) δ 152.57, 141.09, 50.69, 32.78, 25.50, 25.08.

### 2.2 General Procedure for the Preparation Method I of Product 4a-4h

Diazioxalimides **2** (3 mmol) was suspended in 20 mL anhydrous toluene at room temperature, and PPh_3_ (1.57 g, 6 mmol) was added in batches. Subsequently, the mixture was stirred at room temperature for 2-3h until complete consumption of the starting materials was monitored by TLC. The reaction mixture was heated at 115°C for about 6 hours. After removal of the solvent under reduced pressure, the residue was purified on silica gel with *n*-hexane/EtOAc (8:2), and the target compounds **4** were obtained in 45-89% yields.

### 2.3 General Procedure for the Preparation Method II of Product 4a-4h

A solution of PPh_3_ (1.57 g, 6 mmol) in anhydrous toluene (8 mL) was added dropwise to a solution of diaziglyoximes **1** (3 mmol) in anhydrous toluene (15 mL) at room temperature over 30 min. After the mixture was stirred for 1 h, the reaction was completed by TLC monitoring, and the resulting iminophosphorane **5** could proceed to the next step without separation. To the reaction mixture were added various aromatic or aliphatic isocyanates (6 mmol) at room temperature, the reaction mixture was stirred for 6 h at room temperature, and the carbodiimides **6** were completely obtained by TLC monitoring. Subsequently, the reaction mixture was heated in an oil bath at 115°C for about 10 h. The solvent was evaporated in a vacuum, and the obtained residual oil was purified by silica gel column chromatography using *n*-hexane/EtOAc (8:1) as the eluent. The product was recrystallized from *n*-hexane/EtOAc (6:1) to afford diamino derivatives of bis-1,2,4-oxadiazole **4a-4f**.

#### 2.3.1 N^5^,N^5'^-diphenyl-[3,3'-bi(1,2,4-oxadiazole)]-5,5'-diamine (4a)

White solid, m.p.246.5-247.5°C; IR (KBr): 3324, 1594, 1497, 1314, 1231, 1155, 1051 cm^-1^; ^1^H NMR (500 MHz, DMSO) δ 8.66 (d, J = 4.0 Hz, NH, 2H), 7.44 (d, J = 6.0 Hz, ArH, 4H), 7.28 (dd, J = 8.7, 4.7 Hz, ArH, 4H), 7.11-6.79 (m, ArH, 2H). ^13^C NMR (125 MHz, DMSO) δ 152.20, 139.38, 128.47, 121.48, 117.84; HRMS(ESI) m/z: calcd. for: C_16_H_12_N_6_O_2_ [M+H]^+^: 321.3120; found 321.31204.

#### 2.3.2 N^5^,N^5'^-di-p-tolyl-[3,3'-bi(1,2,4-oxadiazole)]-5,5'-diamine (4b)

Yellow solid, m.p.255-257°C; IR (KBr): 3269, 1599, 1495, 1377, 1256,1114,1075cm^-1^; ^1^H NMR (500 MHz, DMSO) δ 8.52 (s, NH, 2H), 7.33 (d, J = 7.1 Hz, ArH, 4H), 7.07 (d, J = 7.0 Hz, ArH, 4H), 2.24 (s, CH_3_, 6H); ^13^C NMR (125 MHz, DMSO) δ 152.83, 137.44, 130.66, 129.34, 118.39, 20.52; HRMS(ESI) m/z: calcd. for: C_18_H_16_N_6_O_2_ [M+H]^+^: 349.1335; found 349.1653.

#### 2.3.3 N^5^,N^5'^-bis(4-chlorophenyl)-[3,3'-bi(1,2,4-oxadiazole)] -5,5'-diamine (4c)

Yellow solid, m.p.248-251°C; IR (KBr): 3295, 1590, 1491, 1399, 1235, 1085, 1012 cm^-1^; ^1^H NMR (500 MHz, DMSO) δ 8.97 (s, NH, 2H), 7.70 (s, ArH, 2H), 7.35-7.21 (m, ArH, 4H), 7.03 (d, J = 7.5 Hz, ArH, 2H); ^13^C NMR (125 MHz, DMSO) δ 152.51, 138.72, 128.80, 125.65, 119.99; HRMS(ESI) m/z: calcd. for: C_16_H_10_Cl_2_N_6_O_2_ [M+Na]^+^: 412.1858; found 412.2208.

#### 2.3.4 N^5^,N^5'^-bis(4-fluorophenyl)-[3,3'-bi(1,2,4-oxadiazole)] -5,5'-diamine (4d)

Yellow solid, m.p.227-234°C; IR (KBr): 3293, 1565, 1409, 1296, 1153, 1094, 1012 cm^-1^; ^1^H NMR (500 MHz, DMSO) δ 8.69 (s, NH, 2H), 7.44 (d, ArH, 4H), 7.11 (d, ArH, 4H). ^13^C NMR (125 MHz, DMSO) δ 158.48, 156.59, 152.91, 136.20, 136.18, 120.23, 120.17, 115.51, 115.33; HRMS(ESI) m/z: calcd. for: C_16_H_10_F_2_N_6_O_2_ [M+Na]^+^:379.2826; found 379.2420.

#### 2.3.5 N^5^,N^5'^-bis(3-chlorophenyl)-[3,3'-bi(1,2,4-oxadiazole)] -5,5'-diamine (4e)

Yellow solid, m.p.247-252°C; IR (KBr): 3289, 1550, 1423, 1286, 1095, 1071 cm^-1^; ^1^H NMR (500 MHz, DMSO) δ 8.97 (s, NH, 2H), 7.71 (s, ArH, 2H), 7.41-7.20 (m, ArH, 4H), 7.03 (d, J = 7.5Hz, ArH, 2H). ^13^C NMR (125 MHz, DMSO) δ 152.42, 141.17, 133.39, 130.54, 121.87, 117.95, 116.99; HRMS (ESI) m/z: calcd. for: C_16_H_10_Cl_2_N_6_O_2_ [M+Na]^+^: 412.1858; found 412.2196.

#### 2.3.6 N^5^,N^5'^-bis(4-bromophenyl)-[3,3'-bi(1,2,4-oxadiazole)] -5,5'-diamine (4f)

Yellow solid, m.p.262-263°C; IR (KBr): 3296, 1551, 1487, 1389, 1232, 1110, 1064 cm^-1^; ^1^H NMR (500 MHz, DMSO) δ 8.85 (s, NH, 2H), 7.45-7.40 (m, ArH, 8H). ^13^C NMR (125 MHz, DMSO) δ152.30, 138.99, 131.57, 120.25, 113.41; HRMS (ESI) m/z: calcd. for: C_16_H_10_Br_2_N_6_O_2_ [M+Na]^+^: 501.0937; found 501.1206.

#### 2.3.7 N^5^,N^5'^-bis(4-nitrophenyl)-[3,3'-bi(1,2,4-oxadiazole)]-5,5'-diamine (4g)

Yellow solid, m.p.288°C; IR (KBr): 3367, 1551, 1412, 1327, 1248, 1177, 1112 cm^-1^; ^1^H NMR (500 MHz, DMSO) δ9.70 (s, NH, 2H), 8.23 (d, ArH, 4H), 7.73 (d, ArH, 4H). ^13^C NMR (125 MHz, DMSO) δ152.00, 146.05, 141.87, 125.53; HRMS (ESI) m/z: calcd. for: C_16_H_10_N_8_O_6_ [M+Na]^+^: 433.2957 ; found 433.3274.

#### 2.3.8 N^5^,N^5'^-dicyclohexyl-[3,3'-bi(1,2,4-oxadiazole)]-5,5'diamine (4h)

White solid, m.p.199-201°C; IR (KBr): 3325, 1572, 1436, 1346, 1271, 1186, 1067 cm^-1^; ^1^H NMR (500 MHz, DMSO) δ5.60(s, NH, 2H), 1.73-1.59 (m, 11H), 1.26-1.03 (m, 11H).; ^13^C NMR (125 MHz, DMSO) δ 156.93, 47.79, 33.63, 25.62, 24.74. HRMS(ESI) m/z: calcd. for: C_16_H_24_N_6_O_2_ [M + Na]^+^: 335.3978; found 335.3308.

## 3 RESULTS AND DISCUSSION

Diaziglyoxime **1**, the direct nucleophilic substitution reaction of dichloroglyoxime with sodium azide in DMF, was easily obtained in high yield, according to the literature [22]. Diaziglyoxime **1** was the key intermediate for the two reaction routes in this paper, but they will deteriorate after being placed at room temperature for 1-2 days, so they need to be stored in the refrigerator for up to a week.

In synthesis method **I**, diaziglyoxime **1** was treated with various aromatic or aliphatic isocyanates to obtain diazioxalimides **2a-2h** high yield using chloroform as solvent. The reaction could be completed in about 3 h (Table **1**).

**Scheme 1:**
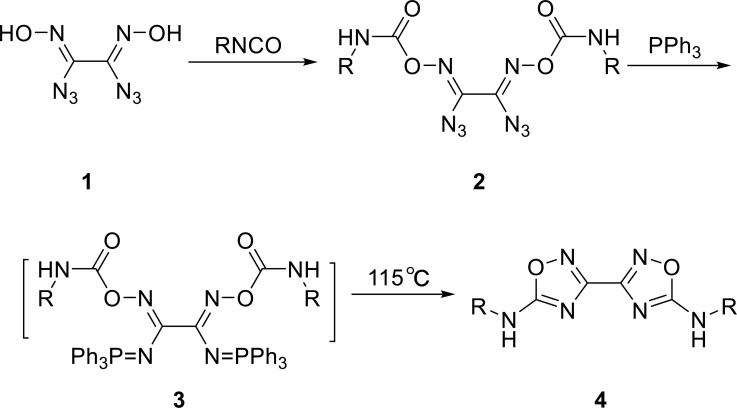
Synthesis method **I** of bis-1,2,4-oxadiazoles **4a-4h**.

Diazioxalimides **2a-2h** are also unstable as a double azide compound. They could not be stored at normal temperature, so the vacuum-drying or Freeze-drying method was used instead of the high-temperature Oven-dry method in the drying process. Subsequently, Diazioxalimides **2a-2h** were easily converted to iminophosphoranes **3** by treatment with Ph_3_P in dry toluene at room temperature, accompanied by the release of nitrogen. Iminophosphoranes **3** are unstable, so they do not need further treatment. The intramolecular aza-Wittig reaction of iminophosphoranes 3 could occur when the reaction was heated at room temperature to 115°C to get the desired target compounds **4** in 72-92% yields (Table **2**). The influence of temperature (from R.T. to 115°C) on the reaction was studied as well. The experimental results showed that the temperature below 100°C greatly prolonged the reaction time, and the reaction was incomplete (Scheme **1**).

**Scheme 2:**
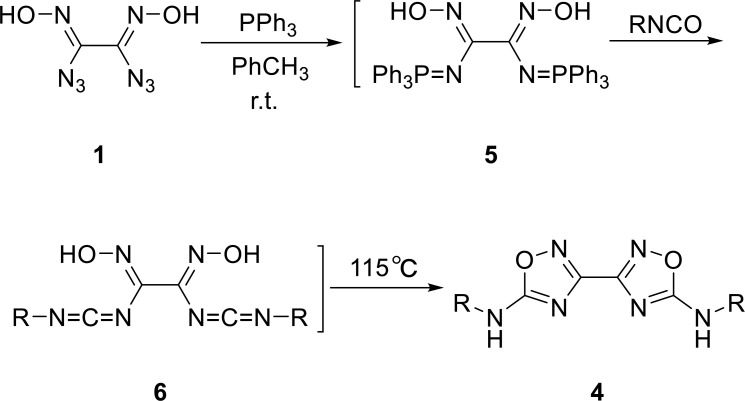
Synthesis method **II** of bis-1,2,4-oxadiazoles **4a-4h**.

In synthesis method **II**, diaziglyoxime **1** could be further designed in the Staudinger reaction and intramolecular aza-Wittig reaction to produce diamino derivatives of bis-1,2,4-oxadiazole **4**. Diaziglyoxime **1** was treated with triphenylphosphonium to produce iminephosphines **5** *via* the Staudinger reaction. Iminephosphine **5** reacted with various isocyanates to obtain carbodiimides **6**. The hydroxyl in molecule **6** was cyclized with carbodiimide by nucleophilic addition to obtain the same target molecule as the first method. We also attempted to separate the intermediate iminophosphorane **5** and carbodiimides **6**; no pure product was obtained because of their stability. The best result was obtained when the reaction was carried out in a one-pot fashion (Scheme **2** and Table **2**).

**Scheme 3:**
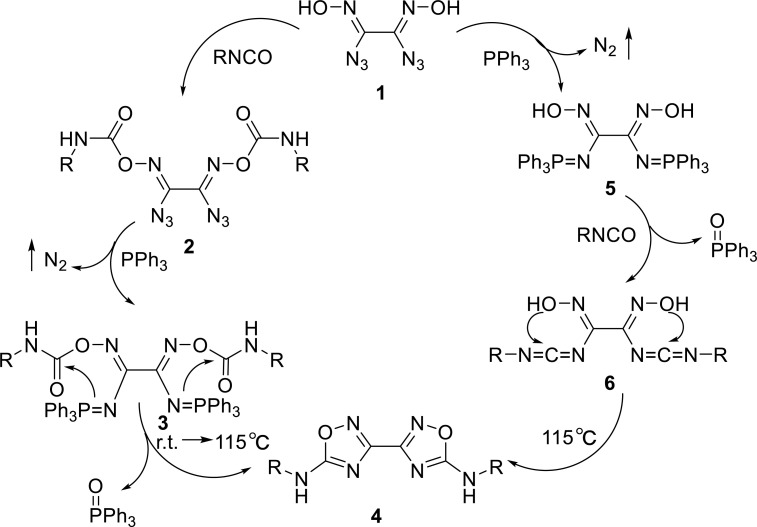
The possible reaction mechanism.

The synthetic mechanism for synthesizing the diamino derivatives of bis-1,2,4-oxadiazole **4** through two different routes is outlined in Scheme **3**. In the synthesis method, **I**, diamino derivatives of bis-1,2,4-oxadiazole **4** were obtained by tandem Staudinger reaction and the intramolecular aza-Wittig reaction of diazioxalimides **2**. In synthesis method **II**, the same target compounds **4** were synthesized *via* tandem Staudinger/intramolecular aza-Wittig/nucleophilic addition cyclization reaction in a one-pot reaction. These two reaction routes were simple and efficient based on a series of highly reliable name reactions.

The structures of **4a-4h** were identified by IR, ^1^HNMR, ^13^CNMR and HRMS. Take **4a** as an example, The IR of **4a** showed N-H at 3324 cm^-1,^ and the characteristic peaks of the benzene ring could also be found at the corresponding positions. The ^1^H NMR showed it could be found the signal for the aromatic protons at d 6.95-7.46 ppm. Furthermore, the structures of **4a** were supported by ^13^C NMR. In addition, as shown in the supporting information, when D_2_O is added to **4a**, the signal of N-H disappears at 8.66 ppm. ^1^H NMR spectrum of **4a** in D_2_O analyses has confirmed the formation of N-H. The supporting information described the preparation and characterization of other compounds **4b-4h** in detail.

## CONCLUSION

In conclusion, we have elaborated two efficient synthesis routes to rapidly construct diamino derivatives of bis-1,2,4-oxadiazole from diaziglyoximes. Readily available starting materials, mild reaction conditions, and simple operating procedures make this protocol highly beneficial for synthesizing bis-1,2,4-oxadiazoles.

## Figures and Tables

**Table 1:** Preparation of diazioxalimides 2a-2h.

**Product 2**	**R**	**Reaction Time (h)**	**Yield (%)^[a]^**
**2a**	Ph	3	82
**2b**	4-CH_3_C_6_H_4_	3	77
**2c**	4-ClC_6_H_4_	3	71
**2d**	4-FC_6_H_4_	3	74
**2e**	3-ClC_6_H_4_	3	62
**2f**	4-BrC_6_H_4_	3	79
**2g**	4-NO_2_C_6_H_4_	3	81
**2h**	cyclohexyl	3	63

**Note:** [a] Isolated yields.

**Table 2:** Preparation method II of bis-1,2,4-oxadiazoles 4a-4h.

**Product 4**	**R**	**Method I**		**Method II**	
		**Reaction Time (h)**	**Yield (%)^[a]^**	**Reaction Time (h)**	**Yield (%)^[a]^**
**4a**	Ph	6	85	10	63
**4b**	4-CH_3_C_6_H_4_	6	92	10	71
**4c**	4-ClC_6_H_4_	6	81	10	65
**4d**	4-FC_6_H_4_	6	83	10	61
**4e**	3-ClC_6_H_4_	6	79	10	59
**4f**	4-BrC_6_H_4_	6	86	10	69
**4g**	4-NO_2_C_6_H_4_	6	82	10	58
**4h**	Cyclohexyl	6	72	10	53

**Note:** [a] Isolated yield.

## Data Availability

Not applicable.

## LIST OF ABBREVIATIONS

HEDMs: High-Energy-Density Materials
KBr: Potassium Bromide
NMR: Nuclear Magnetic Resonance

## SUPPLEMENTARY MATERIAL

Supportive/Supplementary material will be available on the journal’s website. ^1^H, ^13^C spectra of the compounds **4a-h** prepared are available as supplementary material.
