# Supplementary material for: Simple and Efficient Synthesis of Diamino Derivatives of bis-1,2,4-oxadiazole via Tandem Staudinger/aza-Wittig Reaction
Source: Curr Org Synth. 2023 Apr 19;20(6):589–94. doi: 10.2174/1570179420666221006113032 (PMC10258914; doi:10.2174/1570179420666221006113032)

Supplementary Material

Simple and Efficient Synthesis of Diamino Derivatives of bis-1,2,4-oxadiazole *via* Tandem Staudinger/aza-Wittig Reaction

Hai Xie[1,*], Qing-Qing Hu[1], Ya-Li Zhang[1], Xiu-Ting Qin[1] and Lu Li[1]

[1]*College of Chemistry and Chemical Engineering, Shanxi Datong University, Datong, People's Republic of China*

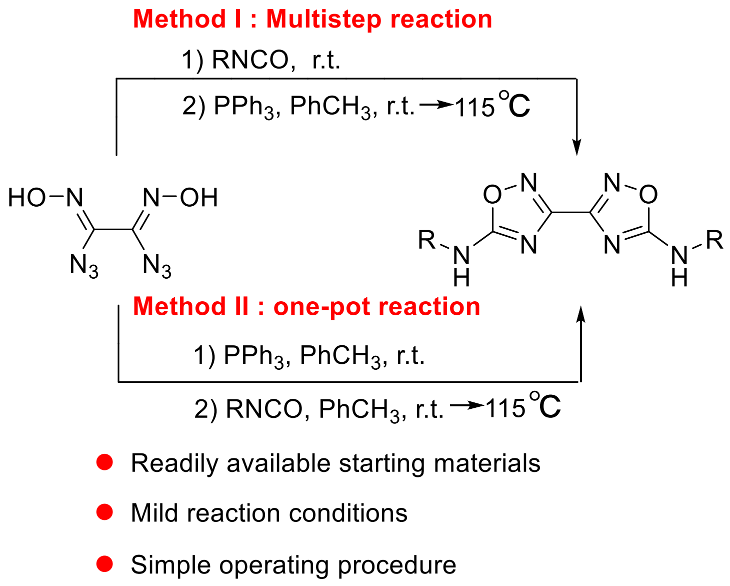

Supporting Information

^{1}H NMR (500 MHz, DMSO) spectra of compound 2a:

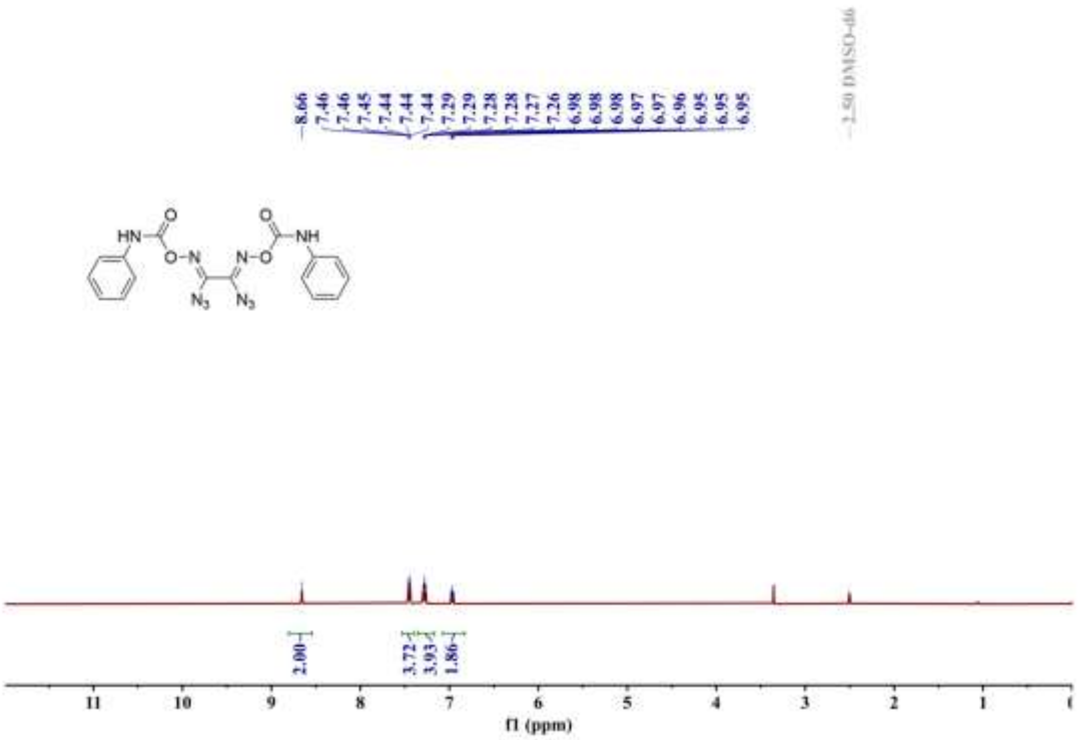

^{13}C NMR (126 MHz, DMSO) spectra of compound 2a:

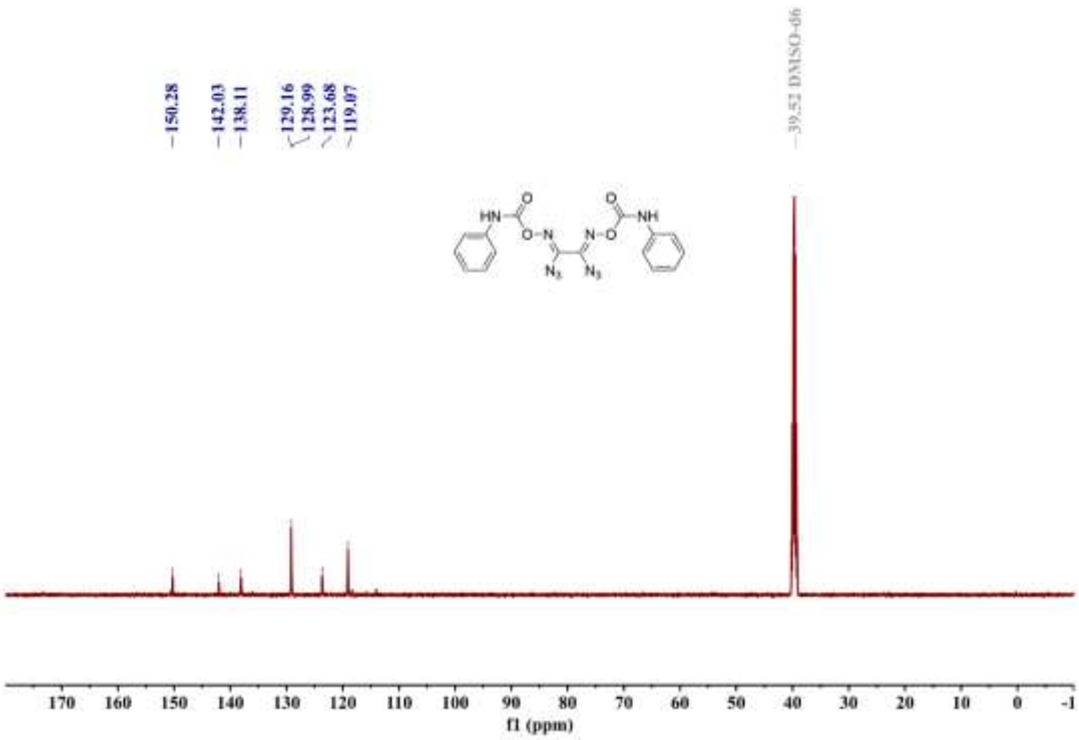

^{1}H NMR (500 MHz, DMSO) spectra of compound 2b:

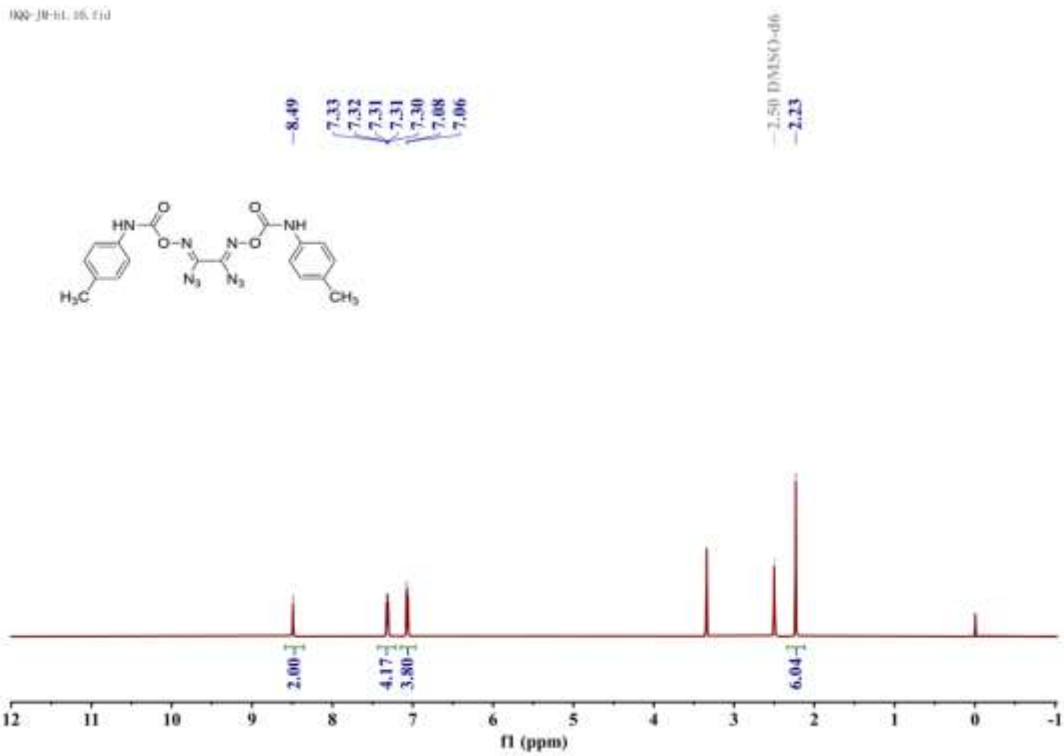

^{13}C NMR (126 MHz, DMSO) spectra of compound 2b:

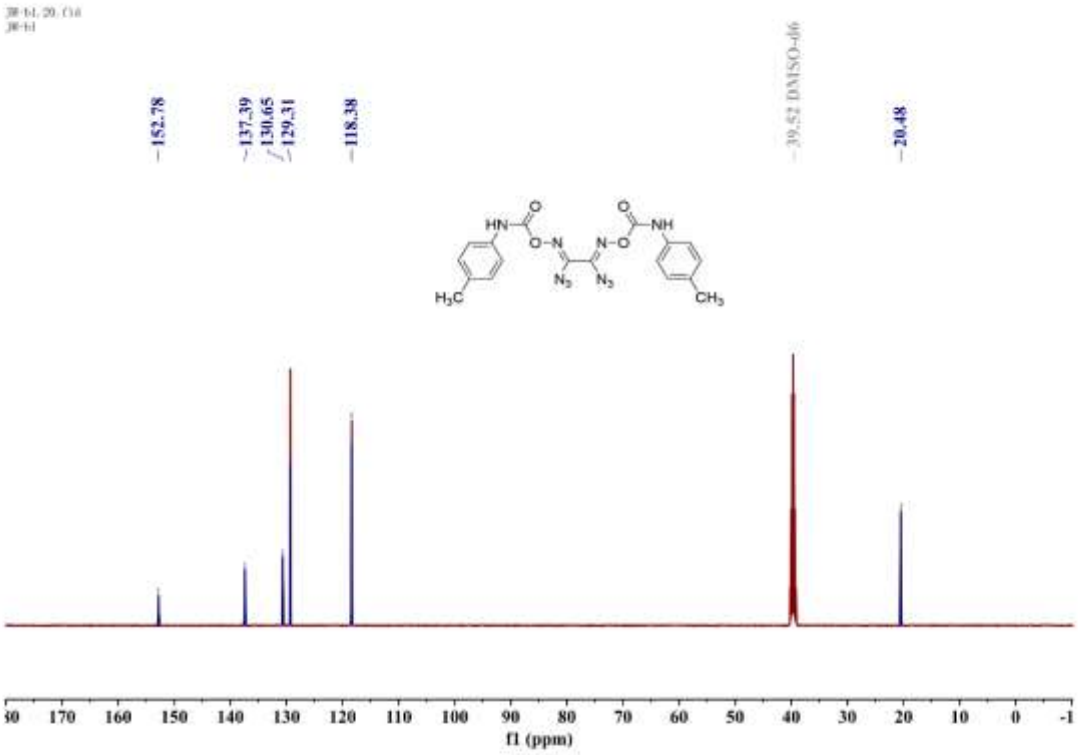

^{1}H NMR (500 MHz, DMSO) spectra of compound 2c:

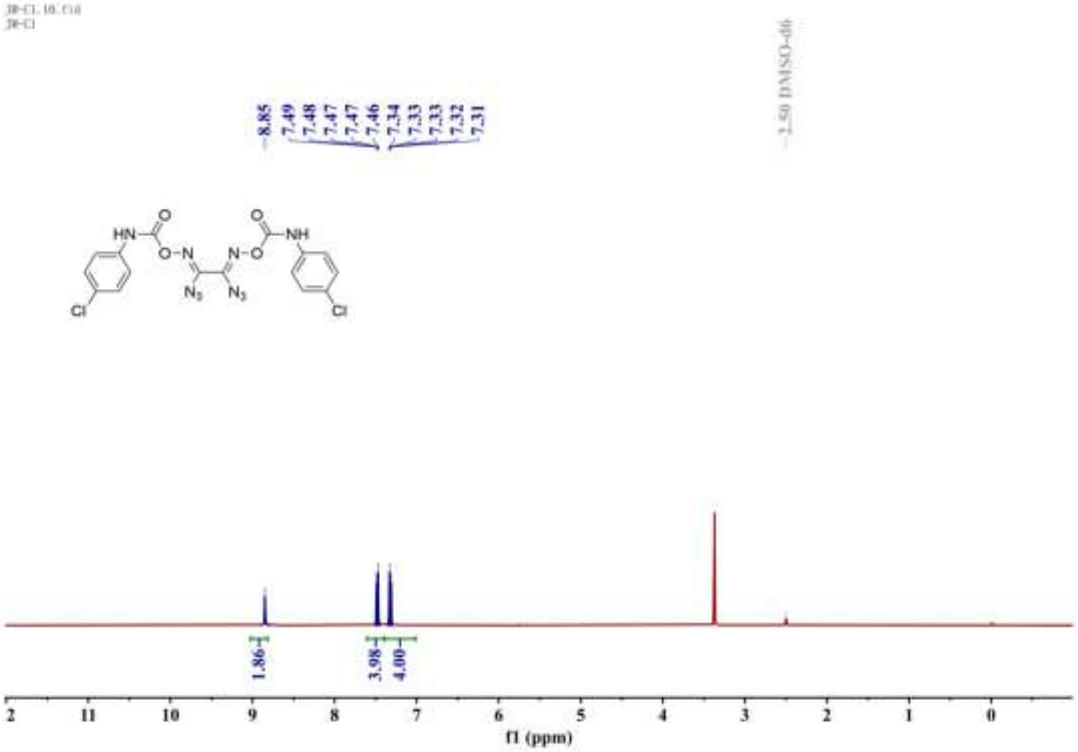

^{13}C NMR (126 MHz, DMSO) spectra of compound 2c:

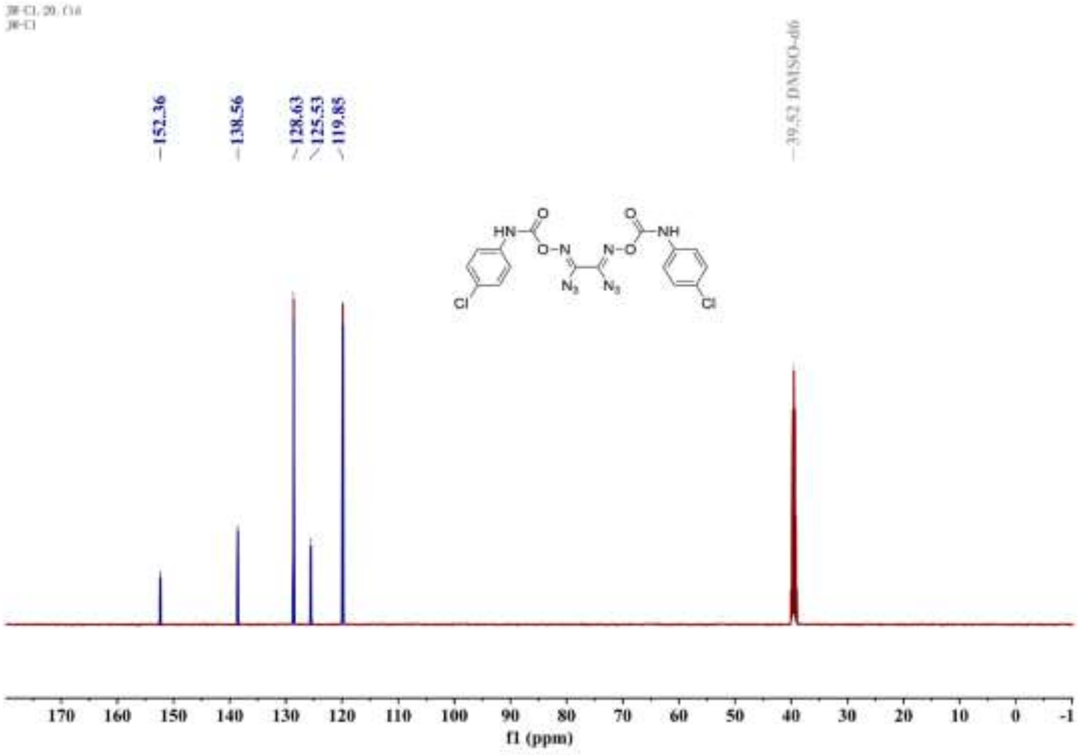

^{1}H NMR (500 MHz, DMSO) spectra of compound 2d:

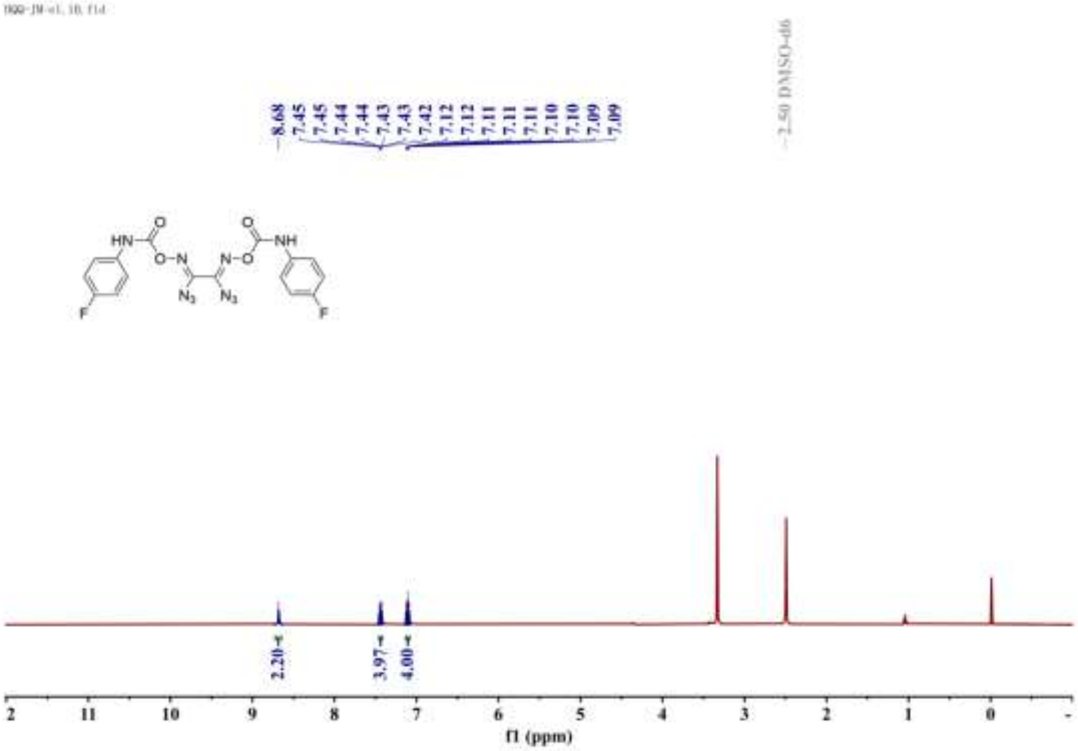

^{13}C NMR (126 MHz, DMSO) spectra of compound 2d:

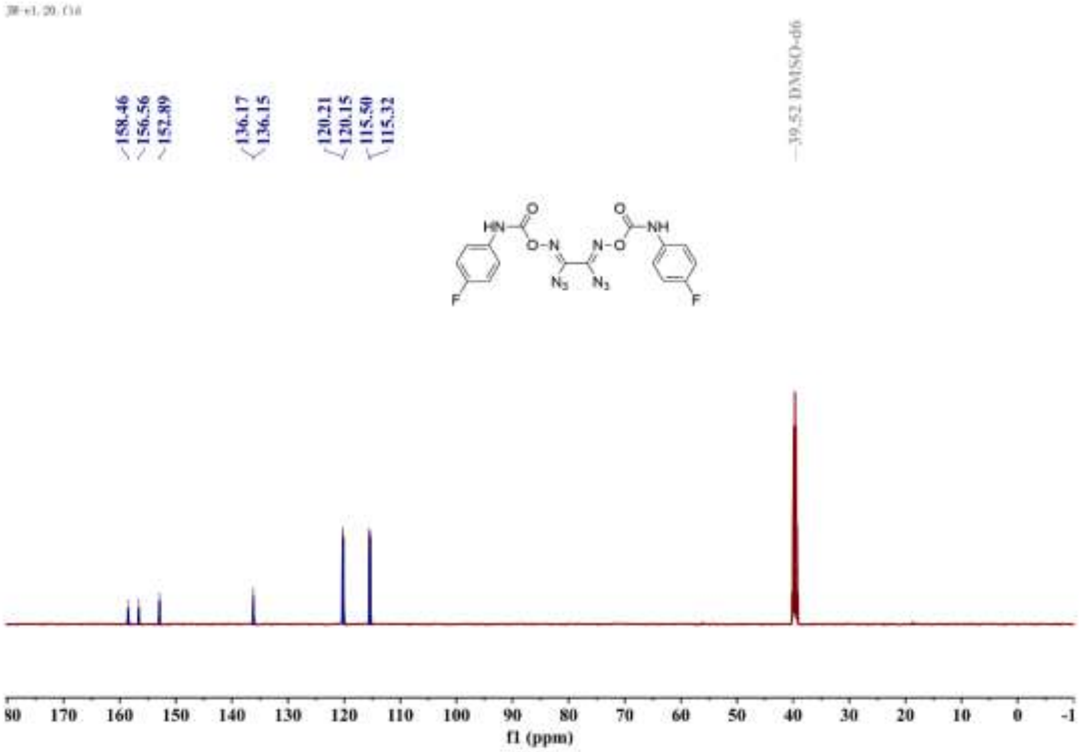

^{1}H NMR (500 MHz, DMSO) spectra of compound 2e:

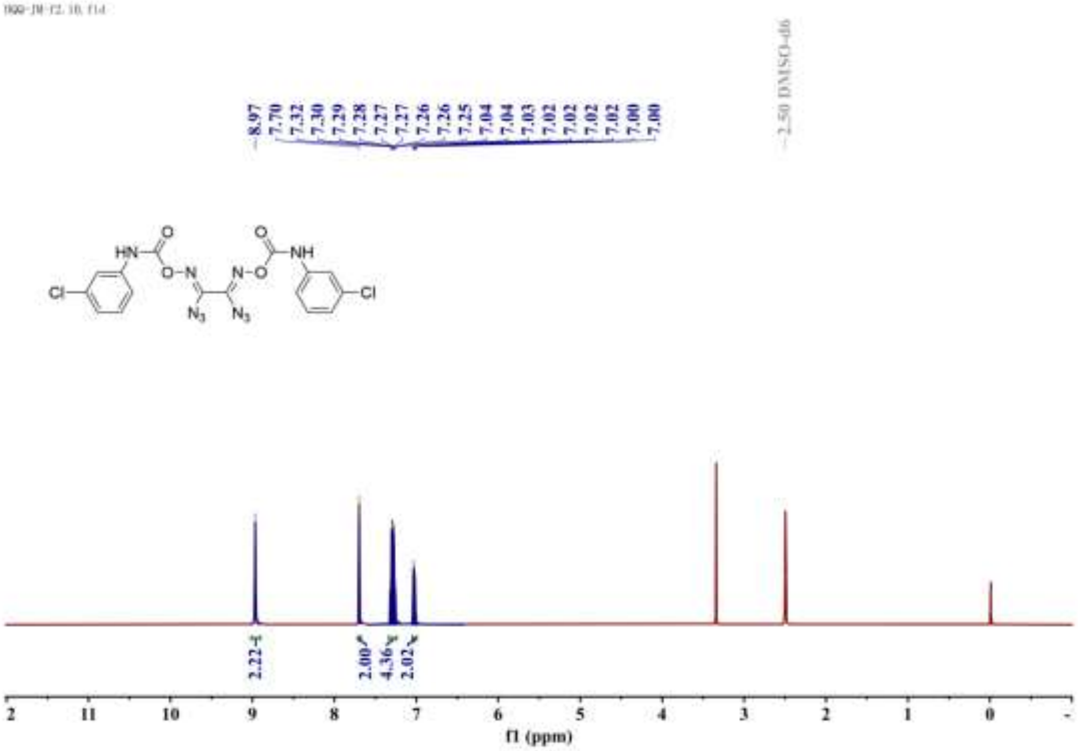

^{13}C NMR (126 MHz, DMSO) spectra of compound 2e:

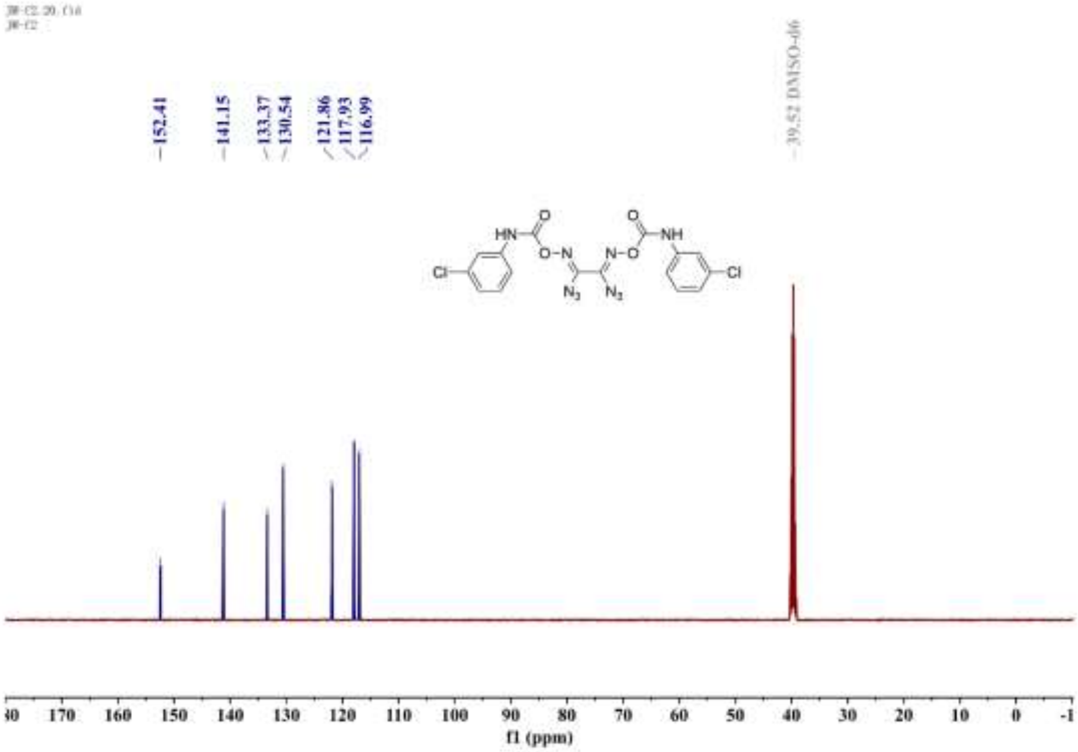

^{1}H NMR (500 MHz, DMSO) spectra of compound 2f:

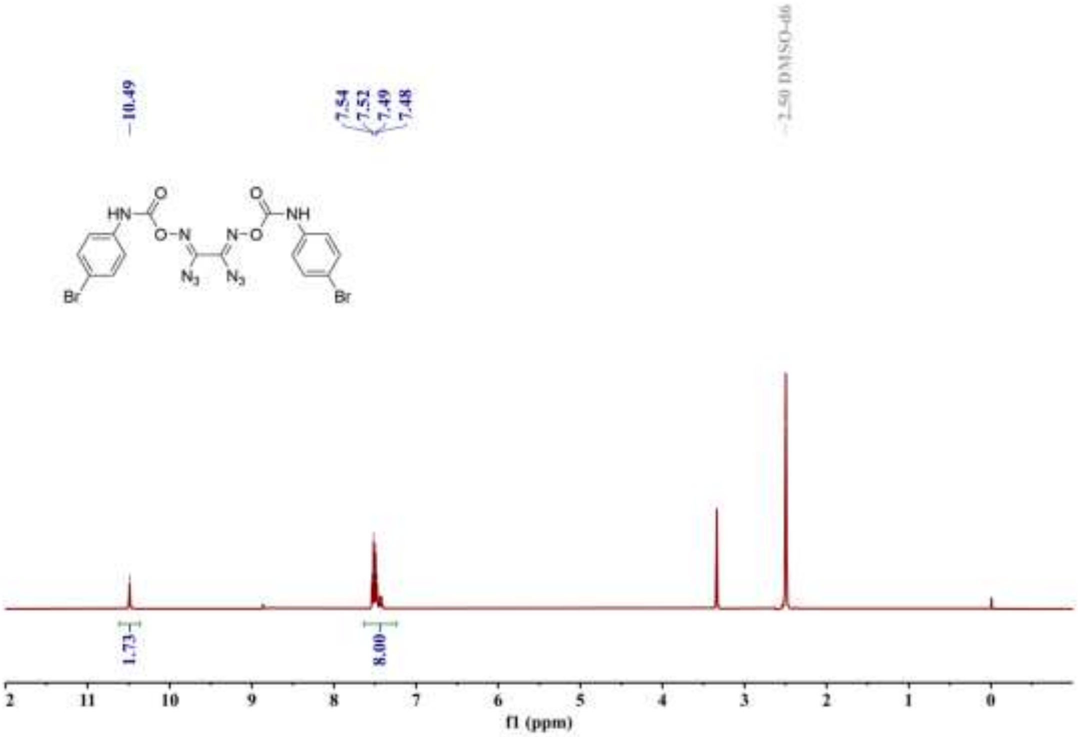

^{13}C NMR (126 MHz, DMSO) spectra of compound 2f:

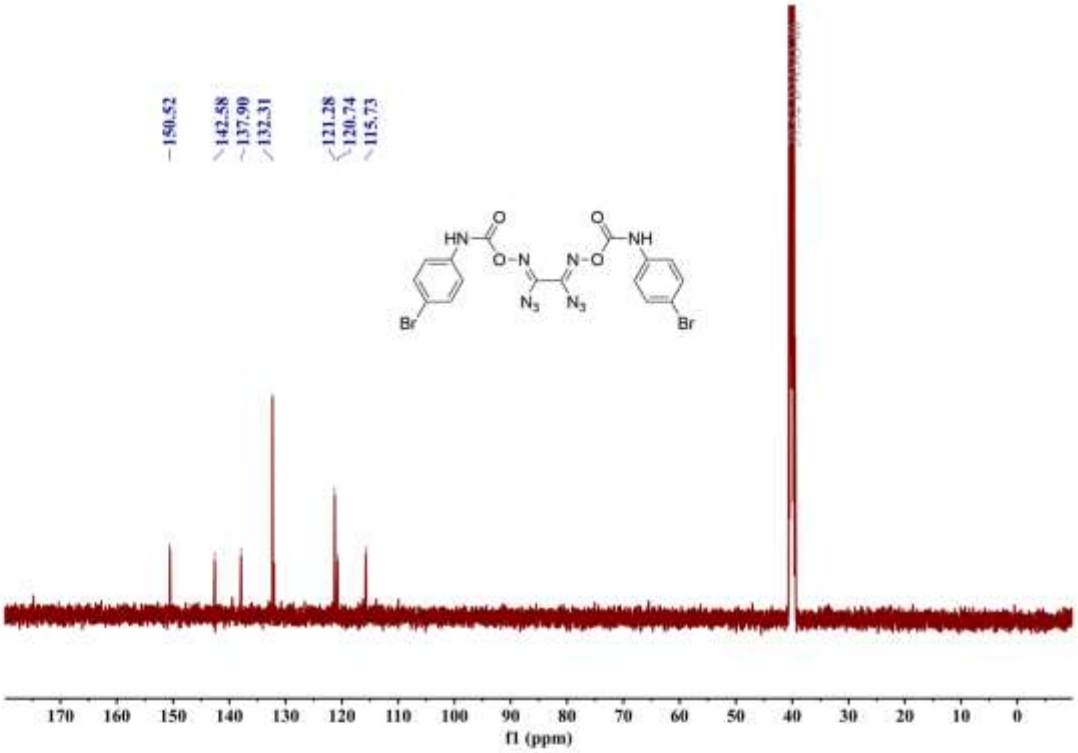

^{1}H NMR (500 MHz, DMSO) spectra of compound 2g:

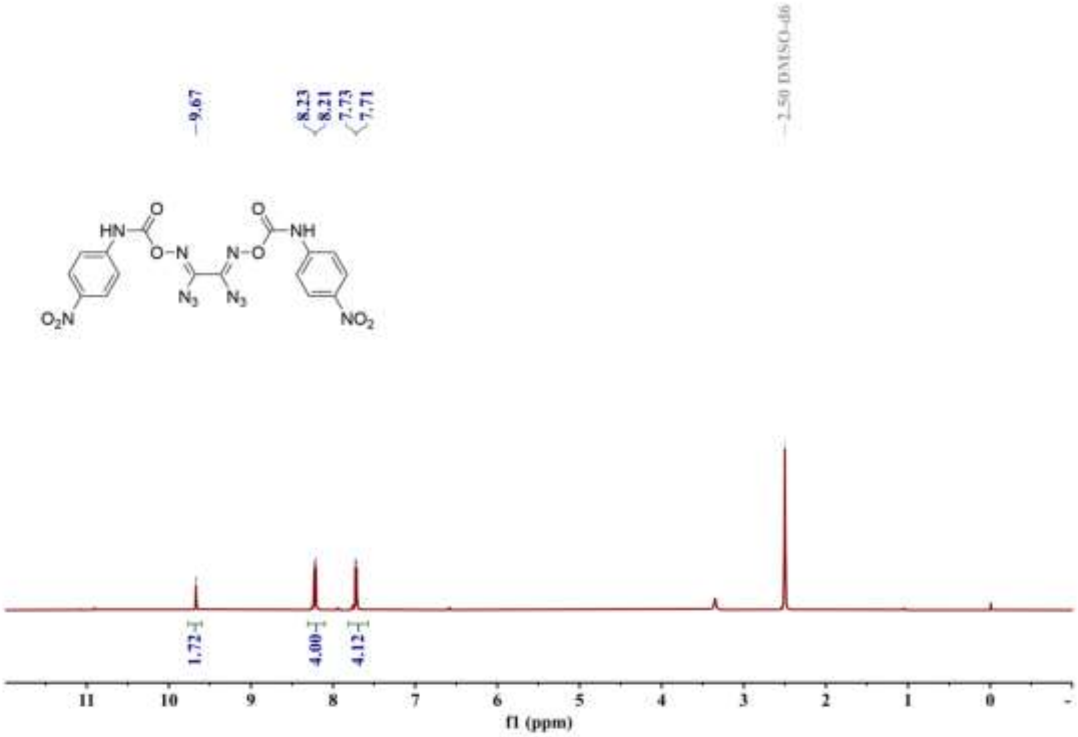

^{13}C NMR (126 MHz, DMSO) spectra of compound 2g:

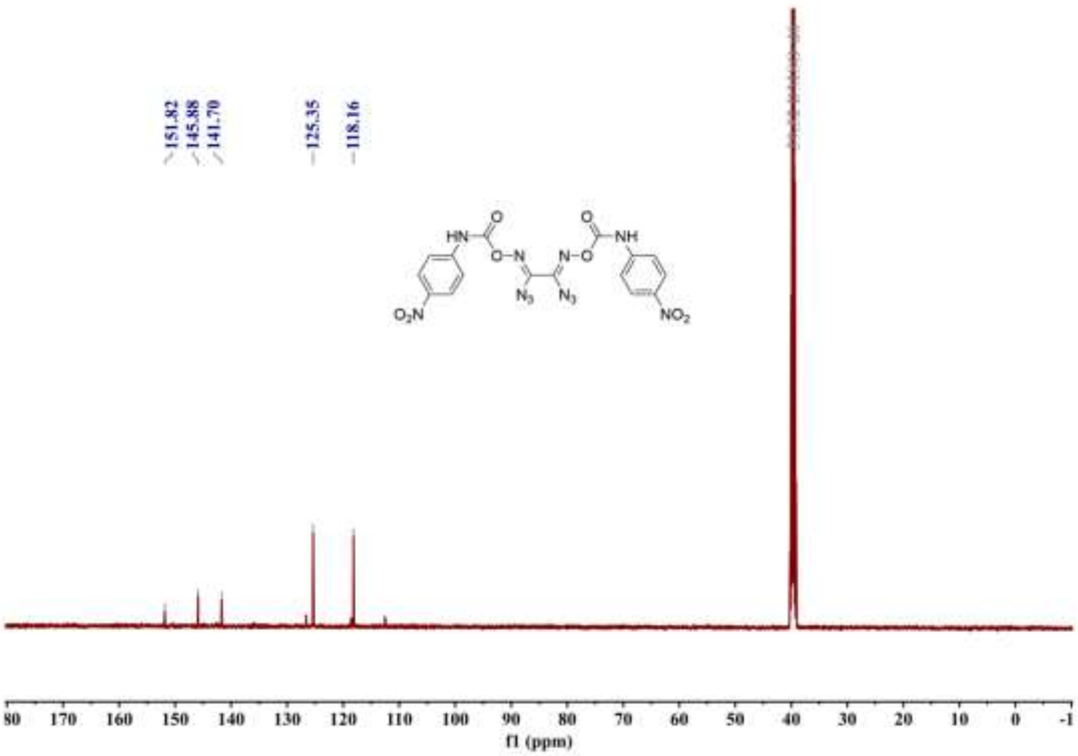

^{1}H NMR (500 MHz, DMSO) spectra of compound 2h:

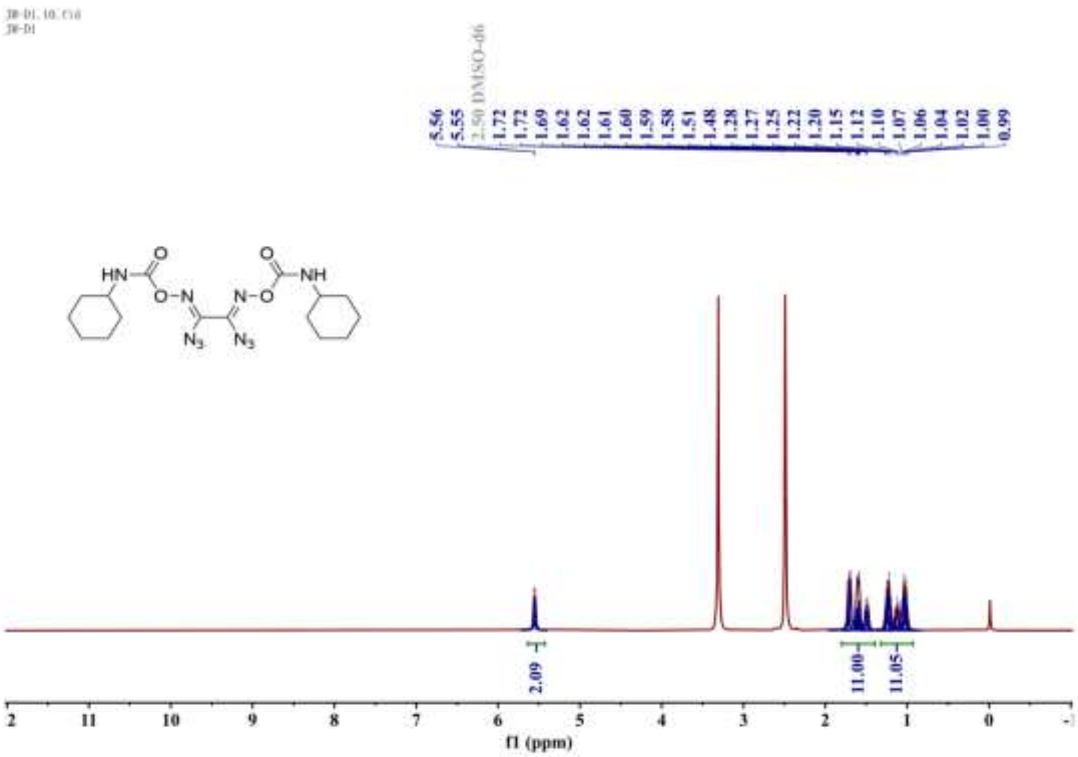

^{13}C NMR (126 MHz, DMSO) spectra of compound 2h:

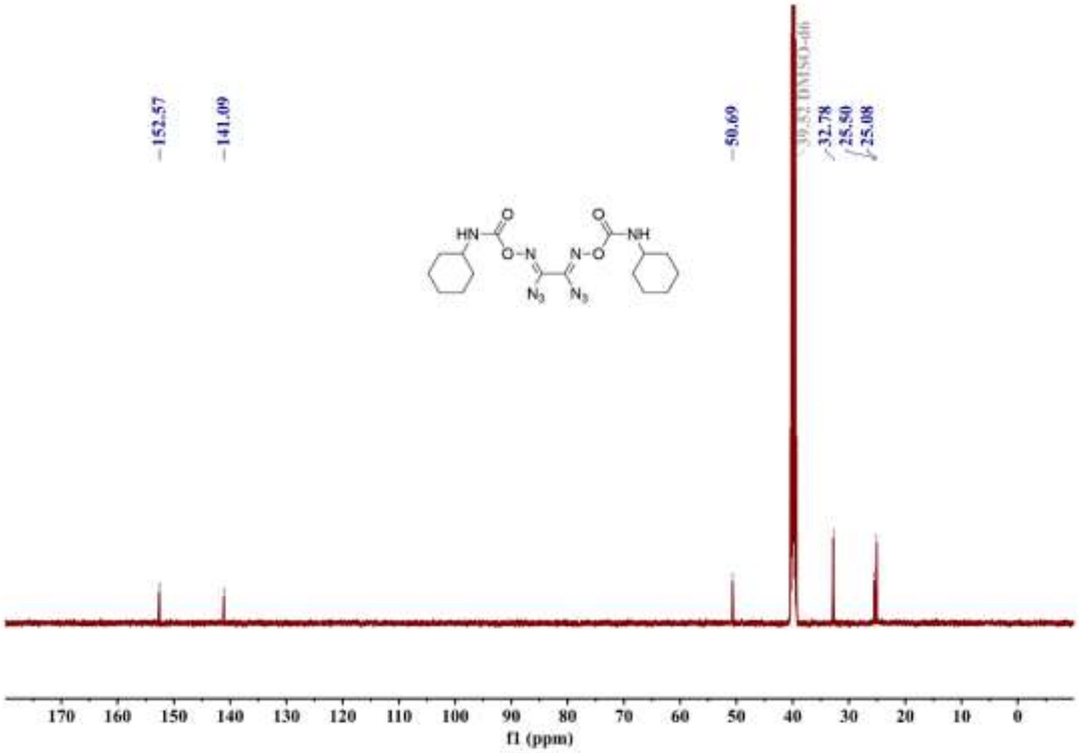

^{1}H NMR (500 MHz, DMSO) spectra of compound **4a**:

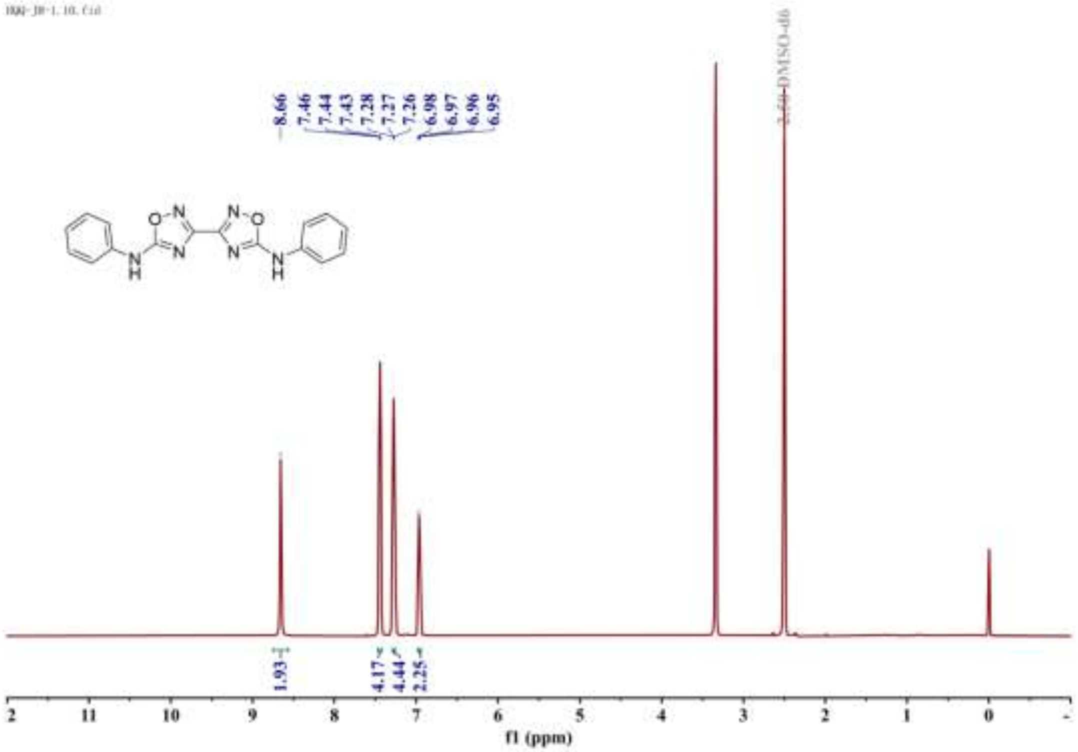

^{1}H NMR (500 MHz, DMSO) spectra of compound **4a(D_2O)**

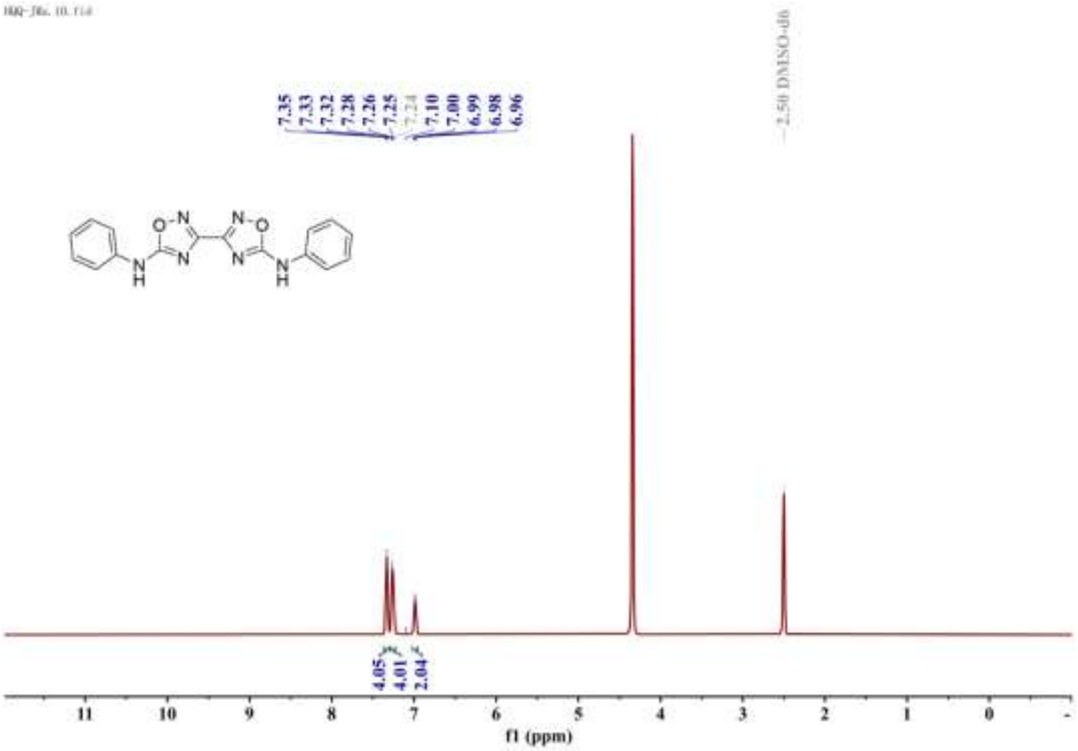

^{13}C NMR (126 MHz, DMSO) spectra of compound 4a:

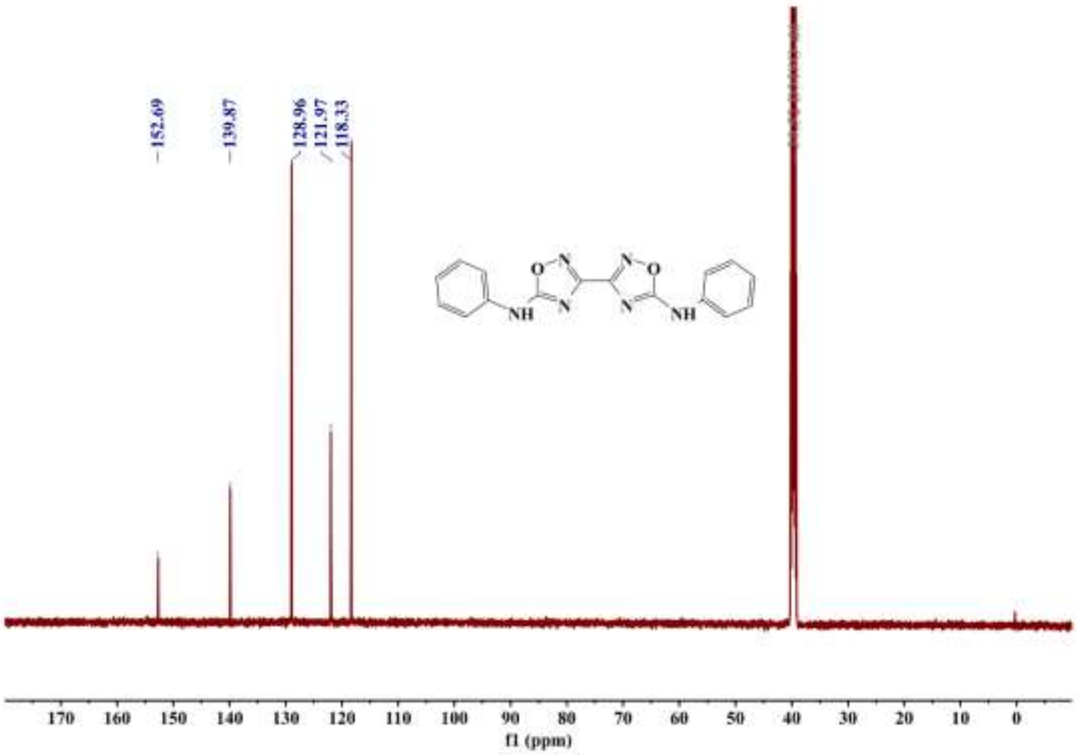

^{71}H NMR (500 MHz, DMSO) spectra of compound 4b:

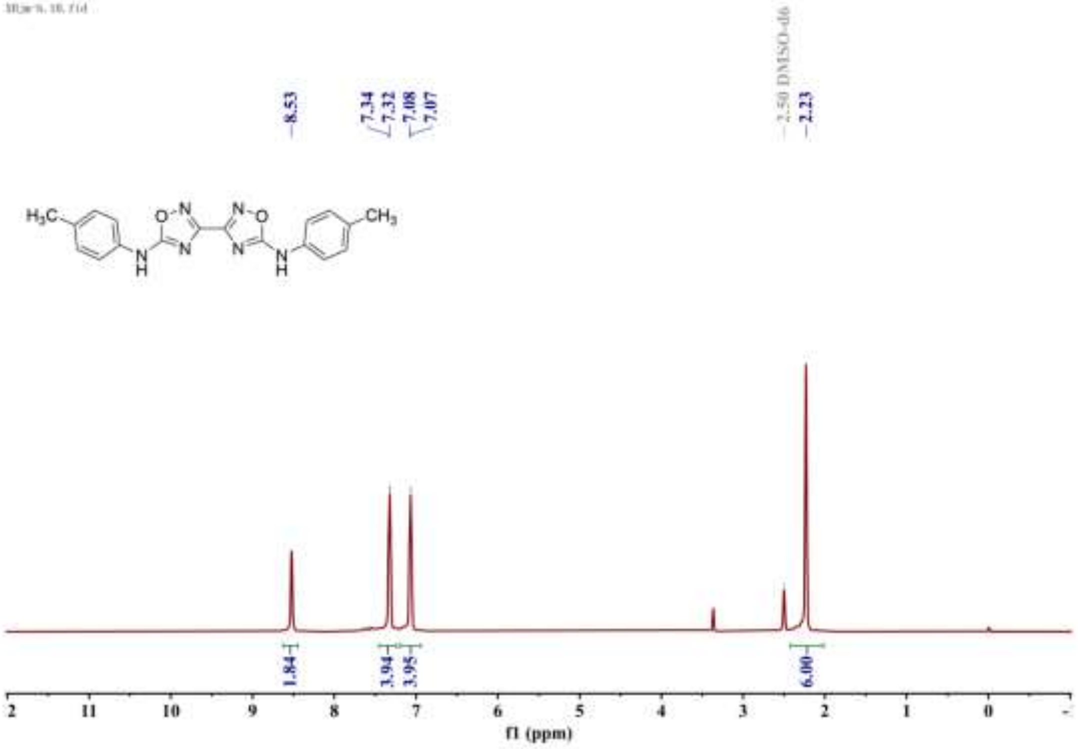

^{13}C NMR (126 MHz, DMSO) spectra of compound 4b:

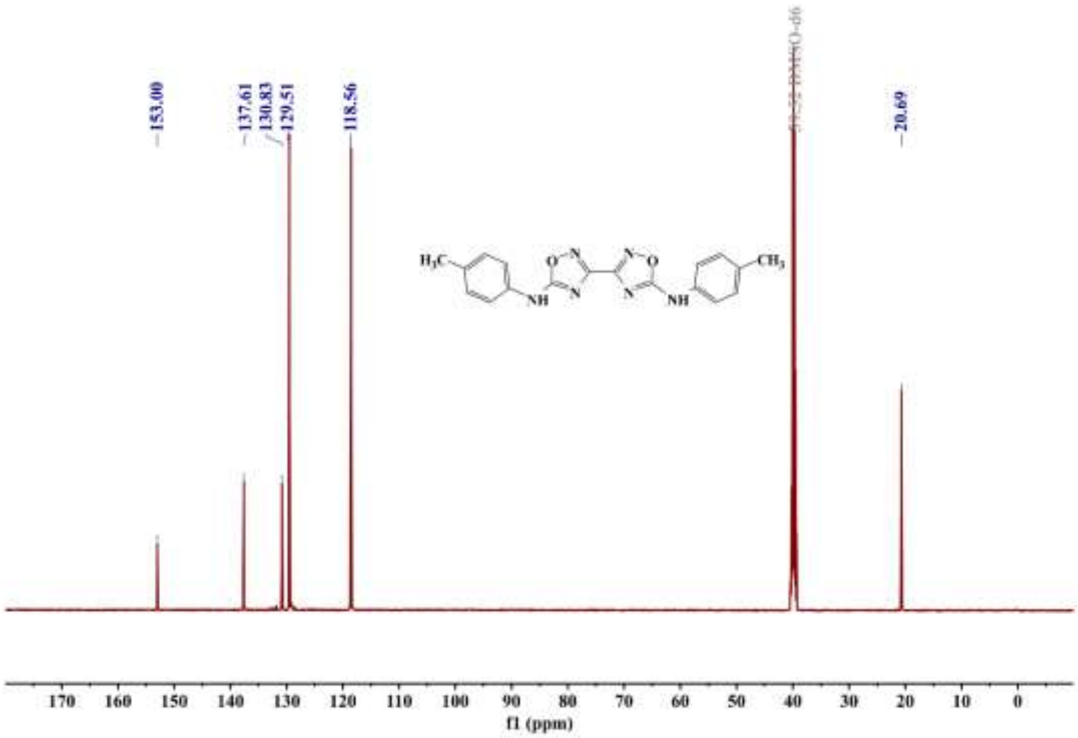

^{1}H NMR (500 MHz, DMSO) spectra of compound 4c:

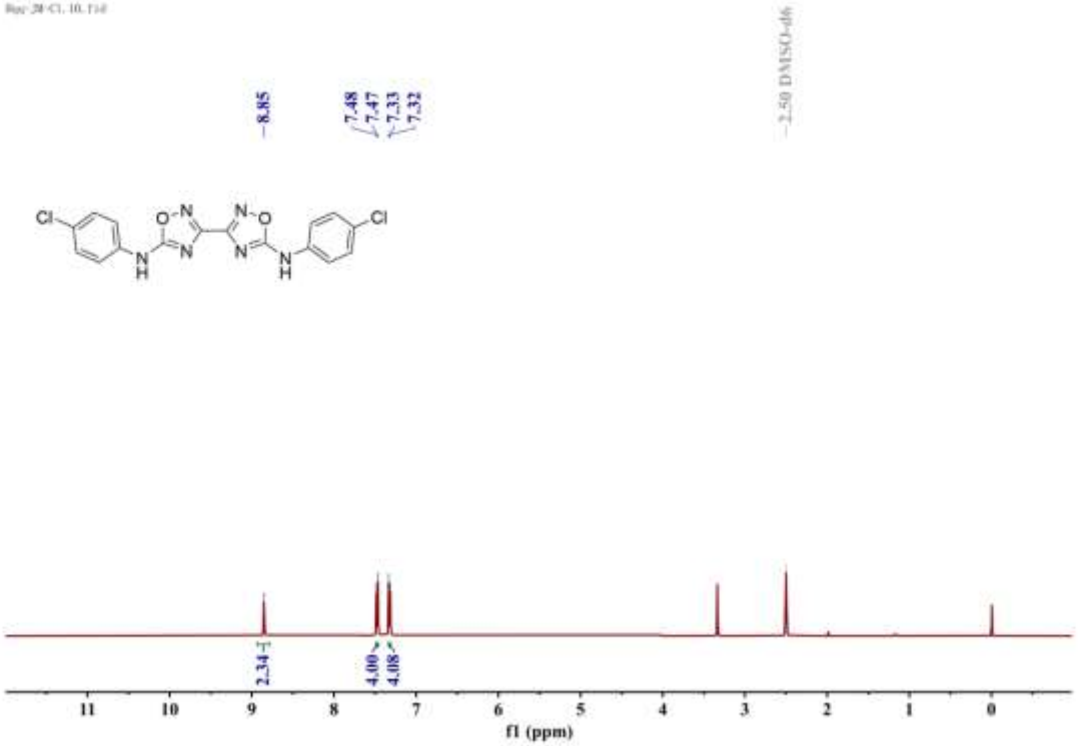

^{13}C NMR (126 MHz, DMSO) spectra of compound 4c:

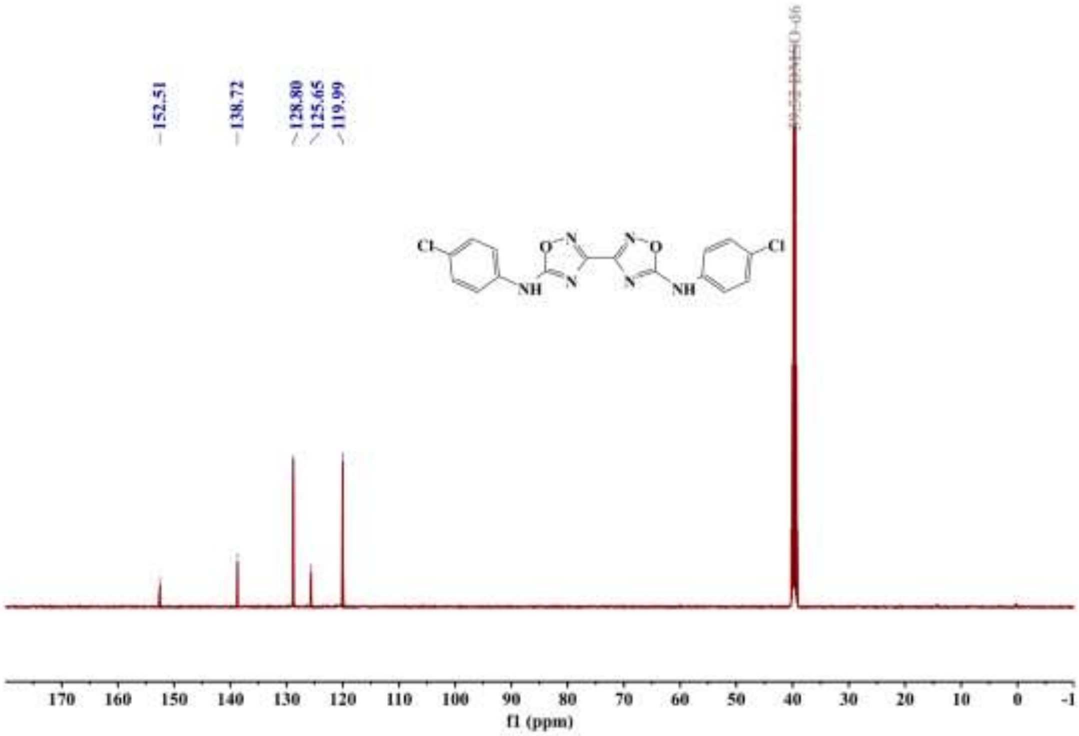

^{1}H NMR (500 MHz, DMSO) spectra of compound 4d:

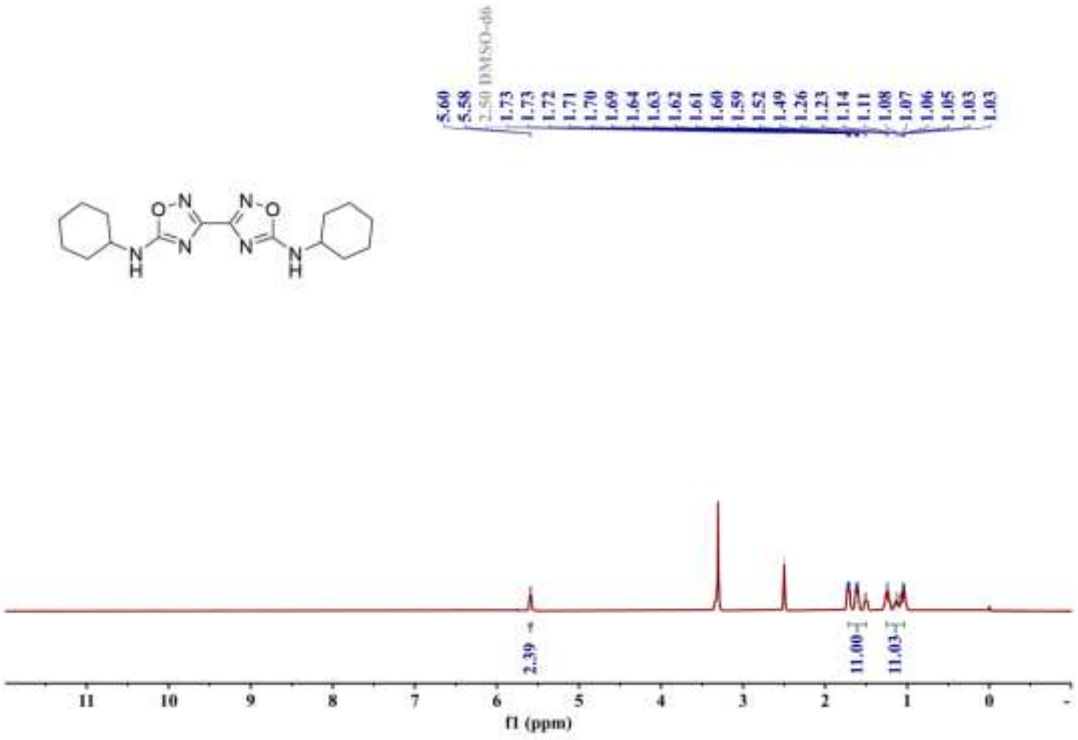

^{13}C NMR (126 MHz, DMSO) spectra of compound 4d:

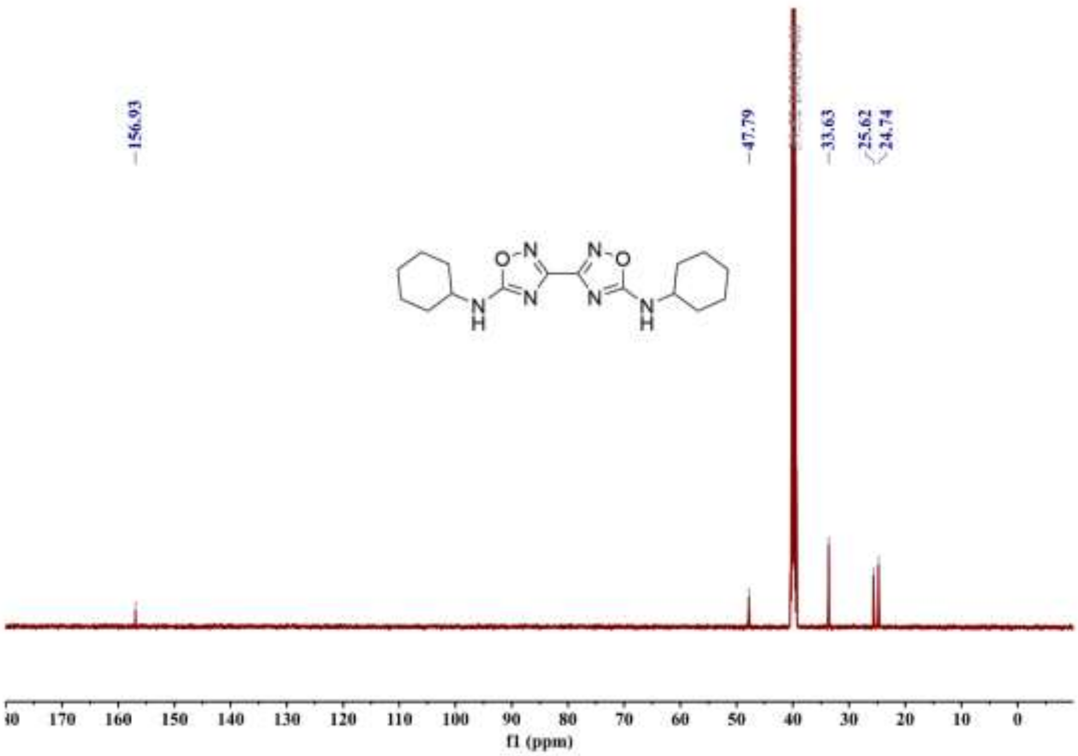

^{1}H NMR (500 MHz, DMSO) spectra of compound 4e:

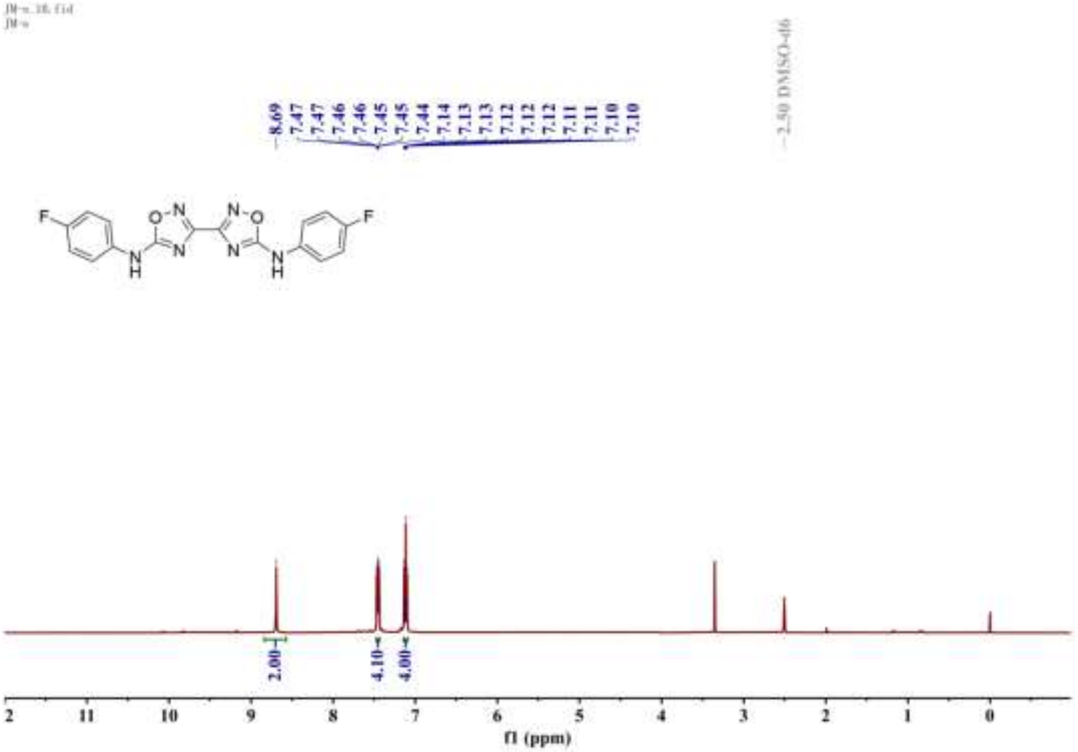

^{13}C NMR (126 MHz, DMSO) spectra of compound 4e:

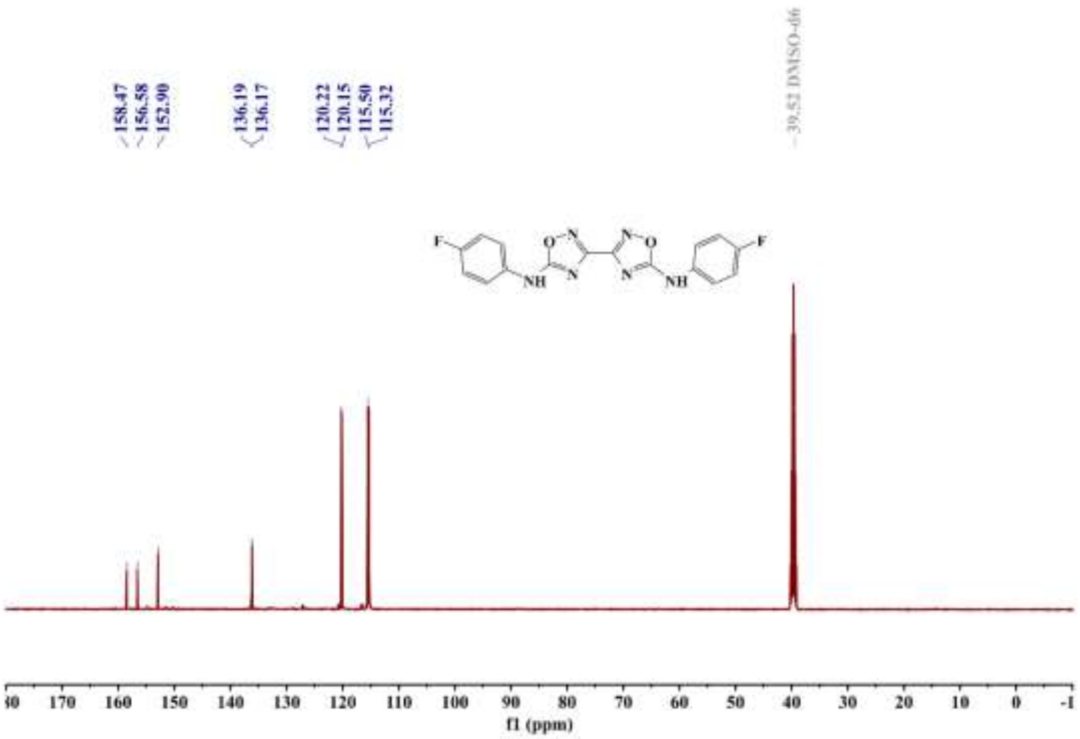

^{1}H NMR (500 MHz, DMSO) spectra of compound 4f:

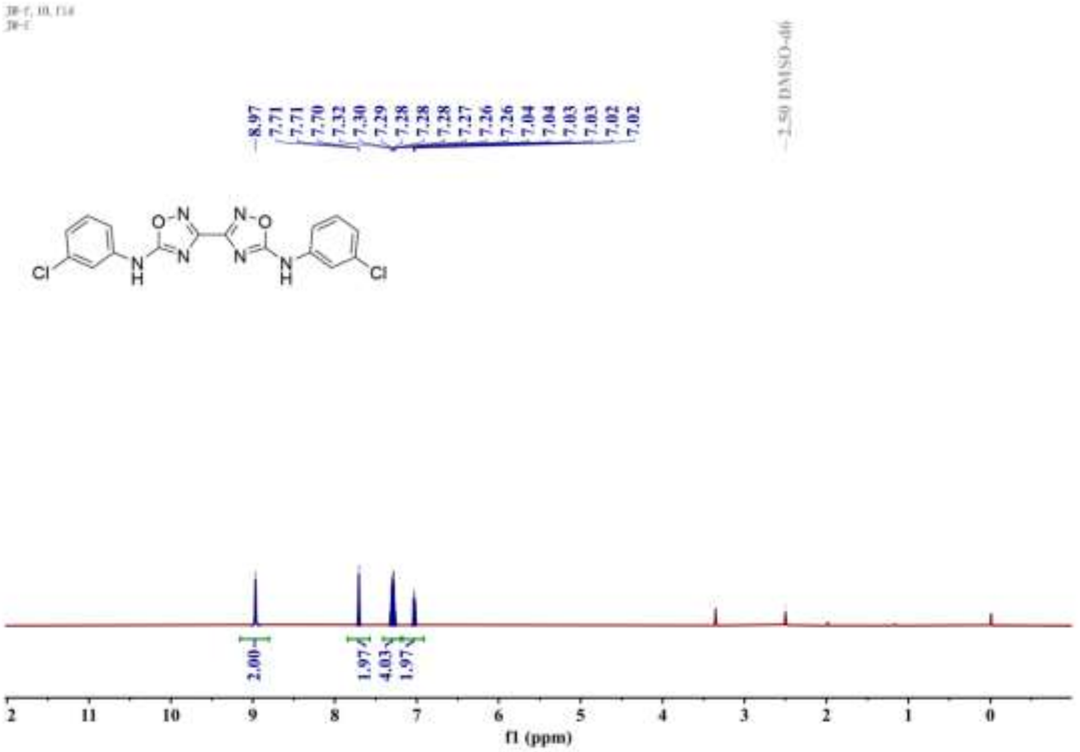

^{13}C NMR (126 MHz, DMSO) spectra of compound 4f:

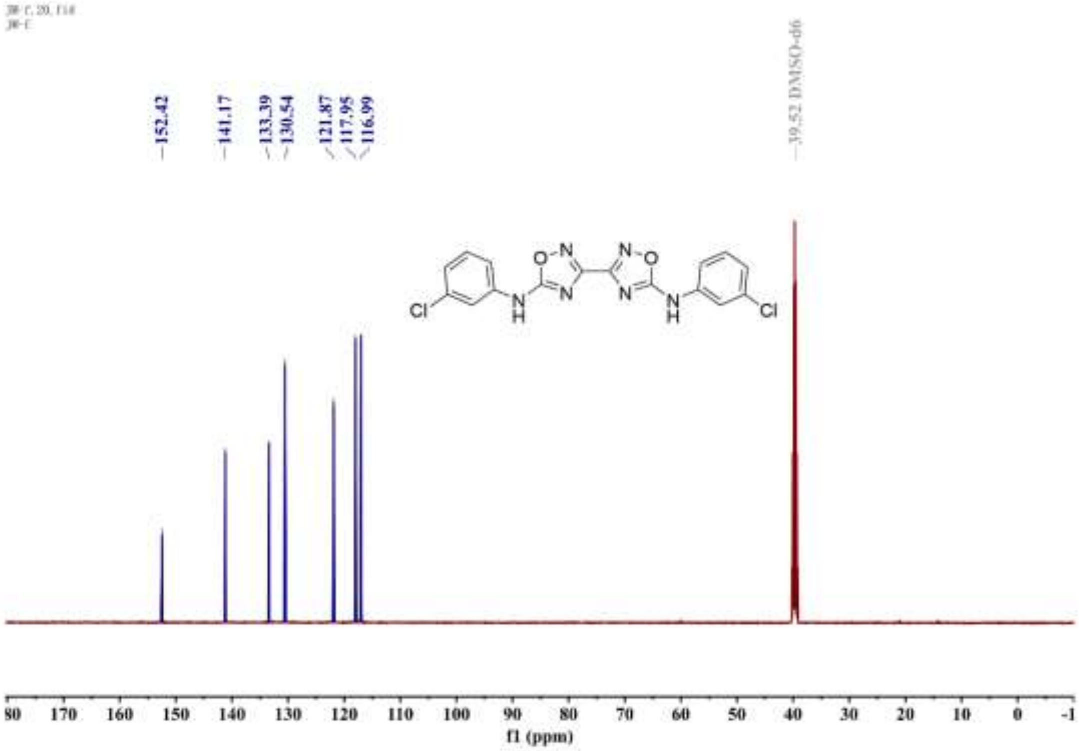

^{1}H NMR (500 MHz, DMSO) spectra of compound 4g:

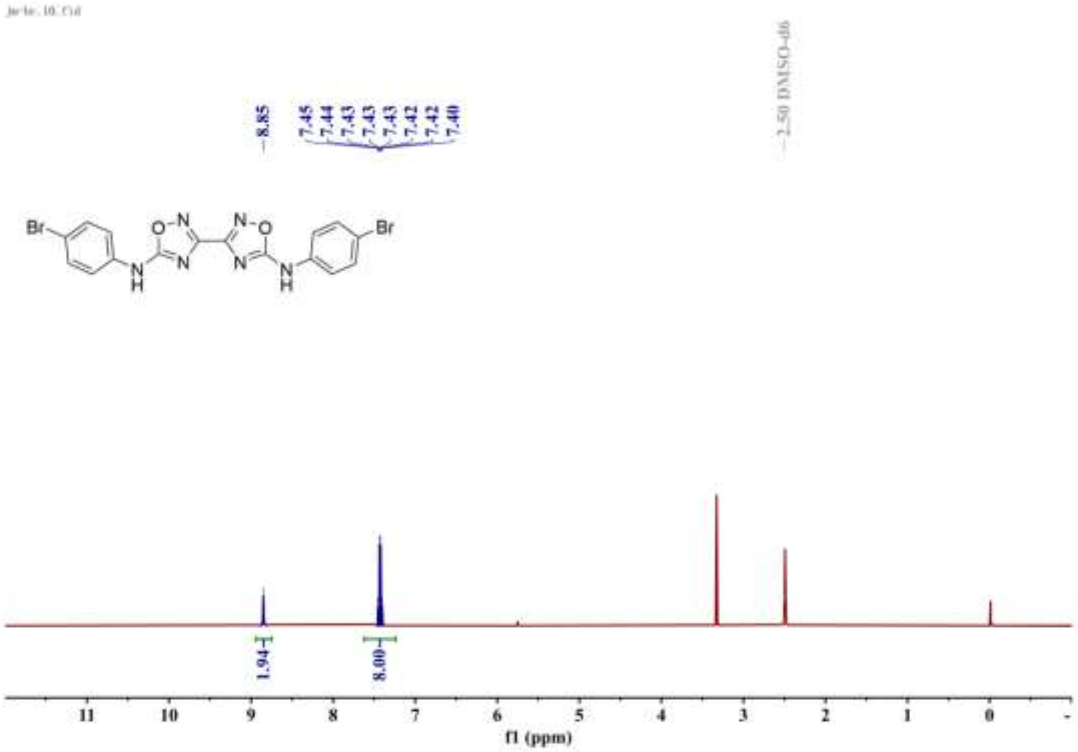

^{13}C NMR (126 MHz, DMSO) spectra of compound 4g:

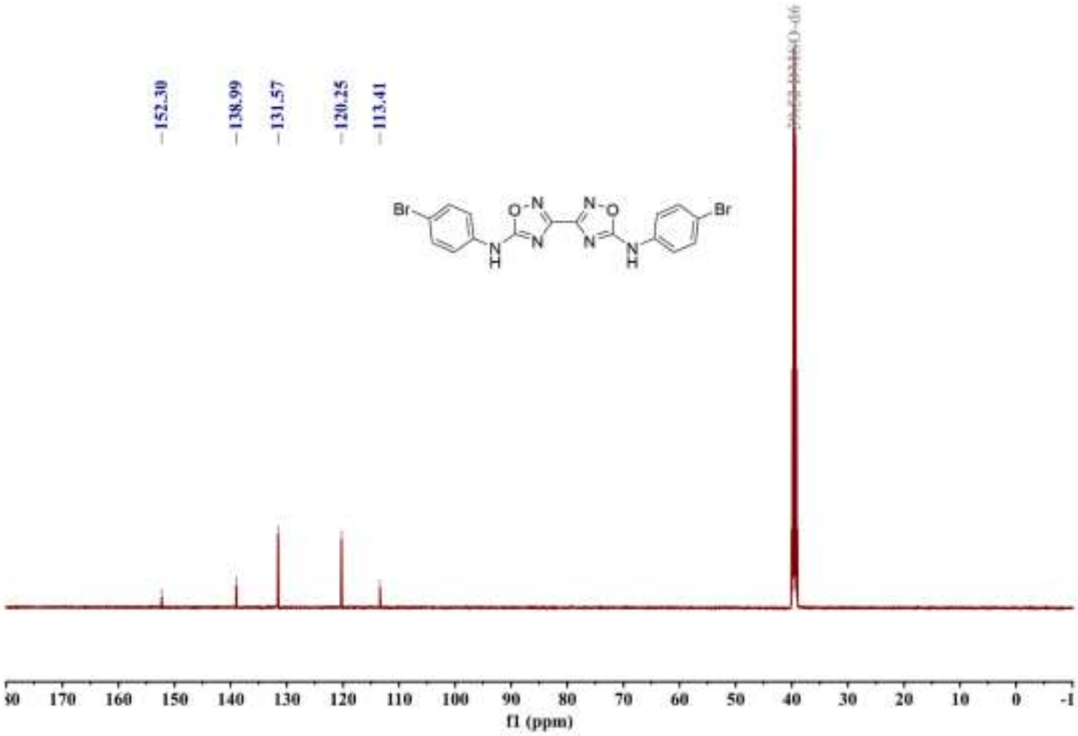

^{1}H NMR (500 MHz, DMSO) spectra of compound 4h:

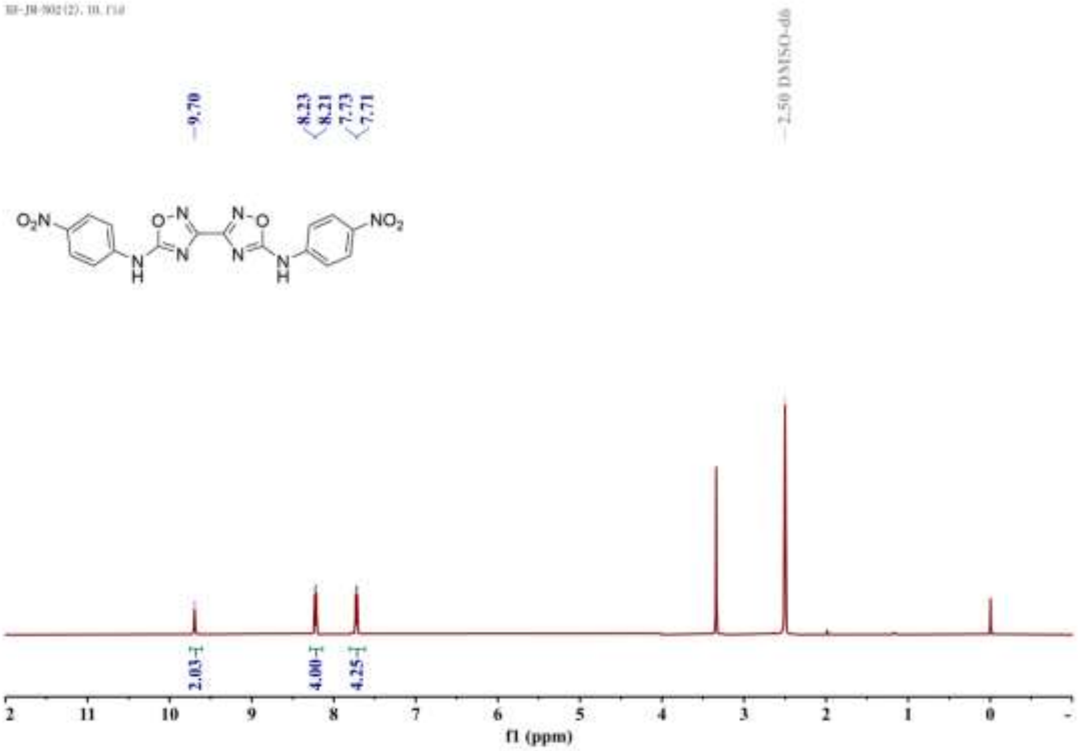

^{13}C NMR (126 MHz, DMSO) spectra of compound 4h:

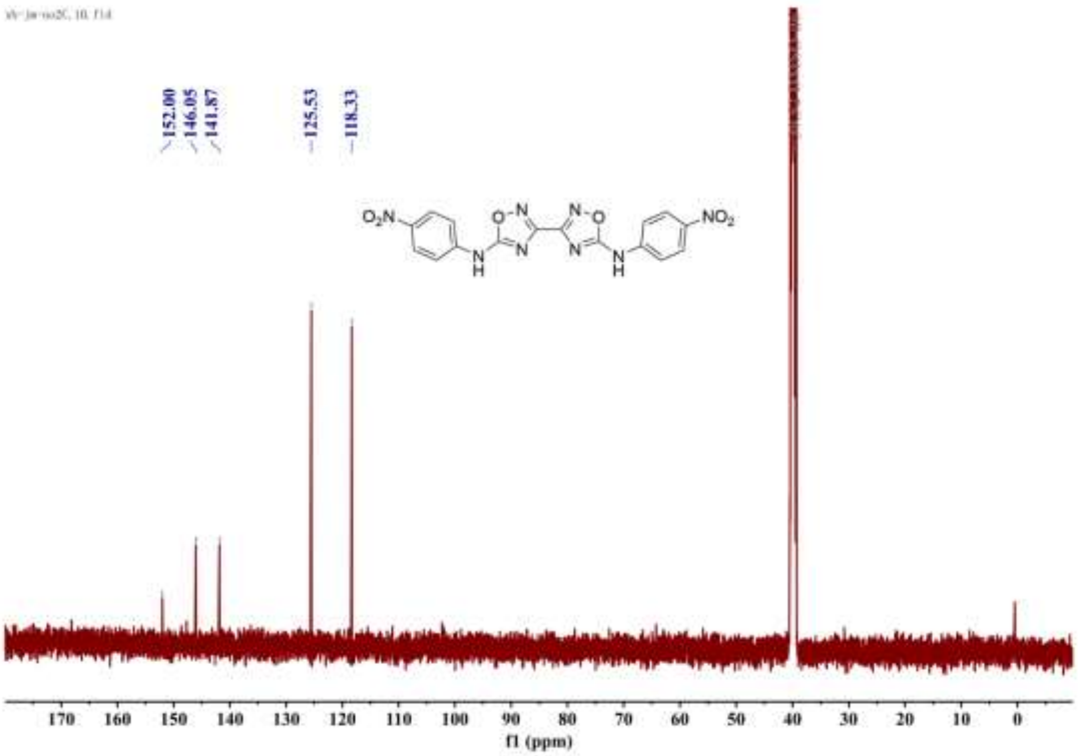

Supplement: Supplementary file 1 — Supportive/Supplementary material will be available on the journal’s website. 1H, 13C spectra of the compounds 4a-h prepared are available as supplementary material. [file COS-20-589_SD1.pdf]
